# Combinations of newly confirmed Glioma-Associated loci link regions on chromosomes 1 and 9 to increased disease risk

**DOI:** 10.1186/1755-8794-4-63

**Published:** 2011-08-09

**Authors:** Tun-Hsiang Yang, Mark Kon, Jui-Hung Hung, Charles DeLisi

**Affiliations:** 1 Bioinformatics Program, Boston University, 24 Cummington Street, Boston, MA 02215, USA; 2 Department of Mathematics and Statistics, Boston University, 111 Cummington Street, Boston, MA 02215, USA; 3 Department of Biomedical Engineering, 44 Cummington Street, Boston University, Boston, MA 02215 USA

## Abstract

**Background:**

Glioblastoma multiforme (GBM) tends to occur between the ages of 45 and 70. This relatively early onset and its poor prognosis make the impact of GBM on public health far greater than would be suggested by its relatively low frequency. Tissue and blood samples have now been collected for a number of populations, and predisposing alleles have been sought by several different genome-wide association (GWA) studies. The Cancer Genome Atlas (TCGA) at NIH has also collected a considerable amount of data. Because of the low concordance between the results obtained using different populations, only 14 predisposing single nucleotide polymorphism (SNP) candidates in five genomic regions have been replicated in two or more studies. The purpose of this paper is to present an improved approach to biomarker identification.

**Methods:**

Association analysis was performed with control of population stratifications using the EIGENSTRAT package, under the null hypothesis of "no association between GBM and control SNP genotypes," based on an additive inheritance model. Genes that are strongly correlated with identified SNPs were determined by linkage disequilibrium (LD) or expression quantitative trait locus (eQTL) analysis. A new approach that combines meta-analysis and pathway enrichment analysis identified additional genes.

**Results:**

(i) A meta-analysis of SNP data from TCGA and the Adult Glioma Study identifies 12 predisposing SNP candidates, seven of which are reported for the first time. These SNPs fall in five genomic regions (5p15.33, 9p21.3, 1p21.2, 3q26.2 and 7p15.3), three of which have not been previously reported. (ii) 25 genes are strongly correlated with these 12 SNPs, eight of which are known to be cancer-associated. (iii) The relative risk for GBM is highest for risk allele combinations on chromosomes 1 and 9. (iv) A combined meta-analysis/pathway analysis identified an additional four genes. All of these have been identified as cancer-related, but have not been previously associated with glioma. (v) Some SNPs that do not occur reproducibly across populations are in reproducible (invariant) pathways, suggesting that they affect the same biological process, and that population discordance can be partially resolved by evaluating processes rather than genes.

**Conclusion:**

We have uncovered 29 glioma-associated gene candidates; 12 of them known to be cancer related (*p *= 1. 4 × 10^-6^), providing additional statistical support for the relevance of the new candidates. This additional information on risk loci is potentially important for identifying Caucasian individuals at risk for glioma, and for assessing relative risk.

## Background

Determining the molecular changes that underlie phenotypic distinctions is a major thrust of cell biology. More specifically, identifying the precise DNA alterations in the genes and regulatory regions that underlie predisposition, initiation and progression of tumors is a central theme of biomedical research. Understanding the molecular changes associated with initiation and progression requires tissue samples from the tumor itself which are often difficult to obtain, as well as from a suitable control population. On the other hand, understanding molecular changes associated with predisposition requires only genomic DNA (e.g., from white blood cells) from the target and control populations, which can be obtained relatively readily. In this manuscript we focus on the latter, since that is where the preponderance of available information is. The methods can, however, be easily extended to the study of somatic genomic associations as control tissue samples from the brain become available.

Identifying predisposition involves (i) identification of the approximate genomic location of a change correlating with phenotypic distinction, which is usually done by finding correlative single nucleotide polymorphisms (SNPs), followed by (ii) the identification of genes or promoters in strong linkage disequilibrium with the SNPs, i.e. those that are coinherited. Following this is (iii) a search for mechanisms, such as point mutations, deletions, and translocations, which can be carried out by sequencing identified genomic regions in a sufficiently large number of samples from affected and control populations.

Here we focus on identifying regions and genes that predispose to glioblastoma multiforme (GBM) (i.e. (i) and (ii), above) by conducting a genome-wide association (GWA) study. A number of such studies have already been carried out for GBM and, as is typical for such studies, very few genes have been consistently identified across different populations [1,2].

## Methods

### Populations

#### The Cancer Genome Atlas (TCGA) samples

**Figure 1:**
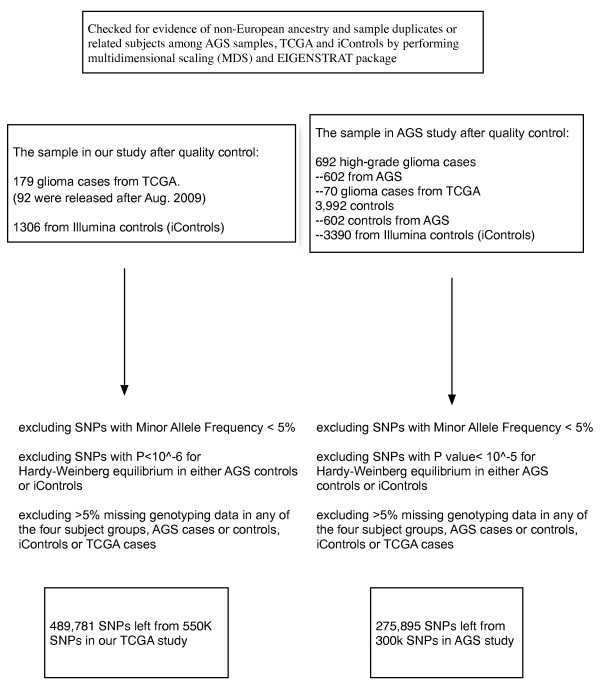
**Subjects and single-SNP exclusion schema for genome-wide association studies**.

To identify risk variants for glioma, we conducted a principal component-adjusted genome-wide association (GWA) study on The Cancer Genome Atlas (TCGA) [3] SNP data. TCGA contains 226 blood samples from glioma patients. Genotypes were determined using the Illumina 550 K HumanHap SNP Array. We eliminated all samples for which more than 5% of the SNPs were missing, and eliminated all SNPs that (i) were determined in fewer than 95% of the samples, (ii) had minor allele frequency less than 5%, or (iii) had a Hardy-Weinberg *p*-value of less than 10^-6^. The procedure is outlined in Figure 1. In order to minimize the confounding effects of ethnicity-specific SNP frequencies in TCGA samples, we confined our study to European Americans, which was the predominant ethnic group in the data. Ethnicity was determined using a two-step screening procedure: (a) self-report of ancestry, and (b) computationally-assisted stratification. The latter was carried out using the EIGENSTRAT package [4]. After this screening, 179 TCGA samples remained. Of these, we used for confirmation only the 92 that were released after August 2009, since the majority of prior samples were included in the Adult Glioma Study (AGS) [1].

#### Control Samples

Normal European American blood samples (*n *= 1366) were downloaded from the Illumina iControlDB (iControls) as the comparison group. After screening, 1306 control samples remained. Because the iControl has more than 3000 samples, we treat it as a background population for our TCGA analysis and use it as a control group (that is also used in AGS). Its treatment as a background population means that we would not expect the results to change with another sample from the same background population. As a partial check of this statement, we divided the glioma samples from TCGA into 2 independent samples, conducting a GWA analysis on each using the same iControl population. If the use of a single control created bias, we'd expect overlapping results. In fact the results have no overlap SNP at the 10^-6 ^significance level (Additional file 1).

### Statistical Methods

#### Association Analysis

Association analysis was performed using the EIGENSTRAT package, under the null hypothesis of "no association between the GBM and control SNP genotypes" based on an additive inheritance model [4]. The significance threshold *p *for association needed to be set stringently to allow for the large number of multiple tests in the GWA study. In particular, if we require that the probability of 1 or more false positives be less than 0.05, we must have 1-*e^-Np ^*≤ 0.05 or *p *≤ 0.93 × 10^-7 ^≈ 10^-7 ^with *N*, the total number of SNPs examined, taken as 550,000. At this level, few if any SNPs will be detected for typical glioma population sizes. The alternative is to accept a less stringent *p*-value, and to eliminate false discoveries by seeking confirmation in an independent study.

#### Meta-analysis

Rigorous methods for combining *p*-values from two independent studies were used. Since the AGS study has many more participants than TCGA, we used unequal weights and employed the method of Stouffer. In particular, the fused *p*-value is given by(1)

where we take sample-specific weights (*W_i_*) proportional to the square root of the "total number of individuals" and *Z_i _*= *F*^-1^(1-*p_i_*); *F*^-1 ^(.) is an inverse standard normal CDF (cumulative distribution function). The false discovery rate is estimated as the fused probability multiplied by the total number of SNPs, which was 300,000 in the AGS study.

The procedure for calculating the fused *p*-values begins with lists of SNPs that have *p*-values of less than 0.001 in each population. We walk down this list, calculating a combined *p *value (equation (1)) for each pair, and accept all SNPs for which the false discovery rate (FDR) is less than 0.05 (or equivalently *p*_12 _= 0.05/300,000 = 1.7 × 10^-7 ^; see Table 1 and additional file 2). When *p *exceeds 0.001 in either population, *P*_12 _no longer meets the required threshold, and the walk stops. As a practical matter, the walk can be stopped at more stringent *p *values without changing the main conclusions. In particular stopping AGS at *p *= 10^-5^, and TCGA at 10^-3^, while requiring that *p*_12 _pass the genomic significance level (1.7 × 10^-7^), loses only 2 SNPs (rs12021720 and rs2810424), neither of which adds new genomic regions.

**Table 1:** Concordant SNPs recovered from TCGA and AGS data, and associated genes

SNP	chr	gene	location	left_gene	right_gene	Genes correlated with SNP (R^2^)	AGS (P1)	TCGA (P2)	FDR
rs2736100^+^	5	**TERT**	5p15.33	**SLC6A18**	**CLPTM1L**	NA	5.30E-13	2.66E-04	7.38E-09

rs1412829*^+^	9	**CDKN2A/B**	9p21.3	LOC100130239	LOC729983	**CDKN2A**(1.0); **CDKN2B**(1.0); C9orf53(0.87)	3.40E-08	3.26E-03	1.27E-03

rs2157719	9	**CDKN2A/B**	9p21.3	LOC100130239	LOC729983	**CDKN2A**(0.97); **CDKN2B**(0.97); C9orf53(0.93); RP11-145E5.4(0.97); LOC100130239(0.97)	6.10E-08	8.00E-03	5.40E-03

rs1063192^+^	9	**CDKN2A/B**	9p21.3	CDKN2A	LOC100130239	**CDKN2A**(0.97); **CDKN2B**(0.97); C9orf53(0.93); RP11-145E5.4(0.97); LOC100130239(0.97)	9.20E-08	8.31E-03	7.95E-03

rs4977756^+^	9	**CDKN2A/B**	9p21.3	LOC100130239	LOC729983	**CDKN2A**(0.97); **CDKN2B**(0.97); C9orf53(0.93); RP11-145E5.4(0.97); LOC100130239(0.97)	4.20E-07	1.12E-02	3.90E-02

rs7530361	1	SLC35A3	1p21.2	LOC730081	HIAT1	SLC35A3(1.0); CCDC76(0.95); HIAT1(1.0); LRRC39(0.95); **SASS6**(1.0); BRI3P1(0.95); LOC730081(1.0)	6.50E-07	2.19E-06	4.29E-05

rs501700	1	HIAT1	1p21.2	SLC35A3	**SASS6**	DBT(0.95); RTCD1(0.89); SLC35A3(1.0); CCDC76(0.95); HIAT1(1.0); LRRC39(0.94); **SASS6**(1.0); BRI3P1(0.94); LOC730081(1.0)	7.10E-07	5.99E-06	9.72E-05

rs1920116	3	LRRC31	3q26.2	LRRIQ4	KRT18P43	MYNN(0.89); LRRC31(1); ARPM1(0.85); KRT18P43(1) LRRC34(1)	1.40E-06	2.88E-03	2.81E-02

rs506044	1	**SASS6**	1p21.2	**SASS6**	LRRC39	DBT(1.0); RTCD1(0.89); SLC35A3(0.95); CCDC76(1.0); HIAT1(1.0); LRRC39(1.0); **SASS6**(1.0); BRI3P1(0.95); LOC730081(0.94)	2.10E-06	2.45E-06	1.57E-04

rs640030	1	**SASS6**	1p21.2	HIAT1	CCDC76	DBT(1.0); RTCD1(0.89); SLC35A3(0.95); CCDC76(1.0); HIAT1(1.0); LRRC39(1.0); **SASS6**(1.0); BRI3P1(0.95); LOC730081(0.94)	2.40E-06	2.57E-06	1.86E-04

rs687513	1	**SASS6**	1p21.2	SASS6	LRRC39	DBT(0.95); RTCD1(0.90); SLC35A3(0.94); CCDC76(1.0); HIAT1(1.0); LRRC39(1.0); **SASS6**(1.0); BRI3P1(0.95); LOC730081(0.90)	2.90E-06	3.91E-06	3.03E-04

rs3779505	7	**ITGB8**	7p15.3	**MACC1**	LOC100130234	**ITGB8**(1.0)	3.00E-06	5.67E-04	1.35E-02

Concordant SNPs (FDR < 0.05) recovered from TCGA (*n *= 97) and AGS data (*n *= 692), and associated genes. The blue boldface indicates the genes that are known to be cancer-associated.

+ reported by Shete et, al.

* reported by Wrensch et, al. and validated on Mayo Clinic population

FDR: Bonferroni corrected False Discovery Rate = 3 × 10^5^P_12 _(eq. 1)

#### eQTL Correlation

We use the coefficient of determination, *R*^2^, to identify the genes with strong eQTL correlation with the SNPs, identified by meta-analysis. *R*^2 ^was calculated based on the correlation between gene expression level and SNP genotype, using the "SCAN: SNP and CNV Annotation Database" resource (http://scan.bsd.uchicago.edu/newinterface/index.html). Genes/SNP combinations with *R*^2 ^greater than 0.8 were considered to be strongly correlated.

#### Odds Ratio

The odds ratio for glioma susceptibility is defined and calculated as follows. For any pair or triplet of the above-mentioned risk alleles, define:

*n*_11_: number of individuals with the given risk allele combination in the glioma sample

*n*_12_: number of individuals without the risk allele combination in the glioma sample

*n*_21_: number of individuals with the risk allele combination in the control sample

*n*_22_: number of individuals without the risk allele combination in the control sample

Then the susceptibility odds ratio (*OR*) is given by(2)

We identified 50 SNP pairs and 88 SNP triplets with significant odds ratios after ruling out combinations of SNPs that were within the same chromosome. Statistical analyses were implemented using R (v2.7) and PLINK (v1.07) [5]. Combinations with odds ratios greater than three, along with *p*-values, are shown in Table 2, which also shows that SNP combinations from chromosomes 1 and 9 are associated with the highest relative risk. Results for all possible pair and triplet combinations (including SNPs within 1 Mb of each other) of the 12 SNPs with *OR *> 3 and for which the risk allele occurrence frequency is > 0.05, can be found in additional file 3.

**Table 2:** Pairwise and triplet SNP combinations with odds ratios greater than 3

SNP Combinations			^+^RISK ALLELE	Freq	OR^eq2^	p-value
*rs1412829 (1.58)		^#^rs7530361 (1.89)	11	5.45E-02	3.31	3.58E-07
*rs1412829 (1.58)		^#^rs501700 (1.90)	11	5.51E-02	3.09	1.95E-06
*rs1412829 (1.58)		^#^rs506044 (1.96)	11	5.47E-02	3.23	5.15E-07
*rs1412829 (1.58)		^#^rs640030 (1.95)	11	5.42E-02	3.28	4.30E-07
*rs1412829 (1.58)		^#^rs687513 (1.93)	11	5.52E-02	3.18	7.32E-07
*rs2157719 (1.49)		^#^rs7530361 (1.89)	11	5.51E-02	3.2	6.83E-07
*rs2157719 (1.49)		^#^rs506044 (1.96)	11	5.54E-02	3.12	9.64E-07
*rs2157719 (1.49)		^#^rs640030 (1.95)	11	5.49E-02	3.16	8.12E-07
*rs2157719 (1.49)		^#^rs687513 (1.93)	11	5.59E-02	3.07	1.35E-06
*rs1063192 (1.42)		^#^rs7530361 (1.89)	11	5.60E-02	3.12	1.13E-06
*rs1063192 (1.42)		^#^rs506044 (1.96)	11	5.63E-02	3.05	1.60E-06
*rs1063192 (1.42)		^#^rs640030 (1.95)	11	5.59E-02	3.08	1.35E-06
*rs4977756 (1.60)		^#^rs7530361 (1.89)	11	5.35E-02	4.28	3.14E-10
*rs4977756 (1.60)		^#^rs501700 (1.90)	11	5.44E-02	4.17	5.57E-10
*rs4977756 (1.60)		^#^rs506044 (1.96)	11	5.36E-02	4.18	4.46E-10
*rs4977756 (1.60)		^#^rs640030 (1.95)	11	5.31E-02	4.24	3.66E-10
*rs4977756 (1.60)		^#^rs687513 (1.93)	11	5.41E-02	4.1	6.86E-10
rs2736100 (0.63)	^#^rs7530361 (1.89)	rs1920116 (0.68)	212	5.01E-02	4.3	5.02E-10
rs11823971 (1.45)	*rs1412829 (1.58)	^#^rs7530361 (1.89)	211	5.21E-02	3.04	5.05E-06
rs11823971 (1.45)	*rs1412829 (1.58)	^#^rs506044 (1.96)	211	5.26E-02	3.01	4.67E-06

Numbers in parenthesis are single SNP odds ratios. Last column is the Wald test *p*-value for the odds ratio of the combination. This is an unadjusted *p*-value, with an 0.05 multiple testing adjusted threshold of *p *= .05/(50+88) = 3.6 × 10^-4^. Freq denotes the combined frequency of the given combination in the case and control populations as a whole.

+ Denotes alleles in which significant shifts occur. 11 denotes significant shift in the minor alleles for both SNPs. 212 denotes significant shifts in major, minor major; 211, significant shifts in major, minor, minor.

# denotes SNP on chromosome 1

* denotes SNP on chromosome 9 in gene CDKN2A/2B.

#### Identification of Associated Pathways and Genes

Tumor initiation is associated with alterations in physiological processes that involve sets of genes and allelic variants in any of several such genes, not all revealed in a single population.

The standard method for identifying altered processes is a pathway enrichment analysis, which can be carried out using a single population [6]. In this case, pathways would be identified by showing that the number of significant SNPs/genes occurring in a particular pathway is above chance expectation. The procedure that we describe here extends this methodology to multiple populations. The assignment of a SNP/gene to a particular pathway using a single population is done using a significance threshold which is loose enough to allow multiple assignments from that population, but not stringent enough for an acceptable FDR in the single population. The FDR is brought down to an acceptable level, as described below, when both populations assign the same gene(s) to the same pathway.

The procedure begins as follows: (1) Identify SNPs having a *p*-value < 10^-3 ^in either of the populations. (2) Identify genes that include these SNPs. (3) Assign the genes thus obtained to KEGG [7] pathways.

In any given pathway, genes identified by the AGS SNPs are generally different from those identified by our TCGA SNPs. Because the *p*-value is not stringent (*p *< 0.001), there is a reasonable chance that a number of SNPs, and therefore pathways, are false positives. We reduced the likelihood of false positives by determining whether, for a given pathway, the number of genes that are common to the two datasets is greater than expected by chance. In a particular pathway, if *n*_1 _genes are identified by TCGA data, and *n*_2 _by AGS (all at nominal *p *< 0.001), we calculate the hypergeometric probability of finding at least *n *genes (the observed number) common to the two sets. If we set an FDR = 0.05, we eliminate all pathways for which the hypergeometric probability exceeds 0.05 divided by the number of pathways to which genes were assigned.

## Results

### Significant SNP candidates

Wrensch et al. [1] screened the Adult Glioma Study (AGS) population using *p = *10^-6 ^and inferred 13 SNPs, 3 of which were confirmed in the Mayo Clinic population at a multiple hypothesis corrected *p*-value of 0.0038 (0.05/13). In a similar fashion, we validated 4 of these 13 SNPs in the TCGA dataset: rs2736100 at chromosome 5p15.33, rs1412829 at 9p21.3, and rs7530361 and rs501700 at 1p21.2 (Table 1, boldface). The last two SNPs are reported as validations for the first time. The above confirmation-based method of correction used for multiple hypotheses is an approximation which gives primacy to one of the populations. The difficulty can be illustrated by first screening on the TCGA data set rather than on the AGS dataset. Using *p = *10^-6^, two SNPs are found for the TCGA screen: rs11840214 in gene *EFNB2 *at 13q33 (*p *= 1.08 × 10^-7^) and rs1909486 at 8q24 (*p *= 4.3 × 10^-7^), and neither of these can be confirmed on the AGS population.

Joint rather than sequential analysis of data from two or more populations can increase the power to detect genetic associations [8]. In particular, using equation (1) in Methods, we identified 12 SNPs (Table 1), confirmed by AGS and TCGA at an FDR < 0.05, one of which was previously confirmed by Wrensch et al. [1] and Shete et al. [2]. Of the 11 remaining SNPs, 4 have been reported by Shete et al., and the remaining 7 SNPs are reported for the first time here. The 12 SNPs are distributed over five genomic regions, including chromsomes 5q15.33, 9q21.3, 1p21.2, 3q26.2 and 7p15.3. Two of these, 5q15.33 and 9q21.3, have been reported in previous studies [1,2]. The 12 SNPs are in strong linkage disequilibrium with 25 genes, 8 of which are known to be associated with cancer (indicated in boldface in Table 1). An additional SNP of interest is rs12341266 at 9q32, which has an FDR of 0.06 and is in the glioma associated gene, *RGS3 *(Additional file 2).

#### Odds ratio for combinatory SNPs

The analysis of TCGA and AGS identifies 12 SNPs, 7 of which have not been previously reported. One of the implications of identifying these additional SNPs is that the number of associated gene groups that can be used to estimate the odds ratio increases combinatorially. Consequently we can expect higher prognostic reliability for individuals possessing a combination of risk alleles, although at some loss of population coverage. We consider here all combinations of two and three SNPs, while constraining our choices to SNPs that are more than a mega-base (Mb) apart, in order to minimize redundant (disequilibrated) information. Specifically, the 12 SNPs are divided into 5 groups based on their chromosomal locations. Chromosome 1 has 5 SNPs clustered together within 1 Mb, and chromosome 9 has 4 SNPs within 1 Mb around genes *CDKN2A, 2B*. The remaining 3 SNPs are located on chromosomes 3, 5, and 7.

### Genes Identified by Conserved Pathway Analysis

We identified 49 pathways (Additional file 4) that contain genes associated with loosely defined AGS or TCGA SNPs (*p*-value < 0.001). 13 of them meet the hypergeometric test at a *p-*value of 0.001 (an FDR of 0.05 divided by 49), i.e. pathways that are relevant to both populations. Each of the 13 pathways has one common gene from the two groups (Table 3). Five genes, *FHIT, GABRG3, PRKG1, DCC*, and *ITGB8*, occurred in more than one of these pathways.

**Table 3:** Pathways that contain significant SNPs (*p *< 10^-3^) inferred from both AGS and TCGA samples

PATHWAY*	AGS_SNP	GENE	TCGA_SNP	GENE
Purine metabolism (p = 3.50E-04)**				
Small cell lung cancer (p = 4.35E-04) **				
Non-small cell lung cancer (p = 2.6E-04) **	rs7617530	FHIT	rs13059601	FHIT
Neuroactive ligand-receptor interaction (p = 8.00E-04) **	rs1011455	GABRG3		
	rs4887546	GABRG3		
	rs1011456	GABRG3	rs12904325	GABRG3

Vascular smooth muscle contraction (p = 3.48E-04) **				
Gap junction (p = 1.30E-04) **	rs4400745	PRKG1		
Long-term depression (p = 6.95E-04) **				
Olfactory transduction (p = 3.47E-04) **	rs4466778	PRKG1	rs1922139	PRKG1

Axon guidance (p = 3.91E-04) **			rs11082983	DCC
Pathways in cancer (p = 2.13E-03)			rs11872471	DCC
Colorectal cancer (p = 8.69E-05) **	rs1145245	DCC	rs12604940	DCC

Focal adhesion (p = 1.95E-03)				
ECM-receptor interaction (p = 8.69E-04) **				
Cell adhesion molecules (CAMs) (p = 1.74E-04) **	rs3779505	ITGB8		
Regulation of actin cytoskeleton (p = 1.56E-03)	rs2301727	ITGB8		
Hypertrophic cardiomyopathy (HCM) (p = 1.22E-03)	rs3807936	ITGB8		
Arrhythmogenic right ventricular cardiomyopathy (ARVC) (p = 9.12E-04) **				
Dilated cardiomyopathy (p = 1.04E-03)	rs2158250	ITGB8	rs3779505	ITGB8

* *p *= Probability of the gene overlap in two independent populations. Multiple testing adjusted threshold of *p *= .05/49 = 10^-3^

** Pathways with *p *< 10^-3^

## Discussion

### Genes strongly correlated with SNP candidates

Eight of the 25 genes we identified are known to be cancer-associated. These include *TERT *[1,9,10], *SLC6A18 *[9], *CLPTM1L *[9,10], *CDKN2A/B *[1,11,12], *SASS6 *[13], *ITGB8 *[14], and *MACC1 *[15] (Table 1). Five of the genes, *TERT, SLC6A18, CLPTM1L*, and *CDKN2A/B*, have previously been shown to be associated with glioma by other GWA studies.

Our combined GWA/pathway analysis predicts four additional genes that are identified in the literature as cancer-related. We therefore predict 29 glioma-associated genes, 12 of them known from previous studies to be cancer-related. It is useful to evaluate the probability that as many as 12 cancer-related genes in a set of 29 would be found by chance. If we use the fraction of OMIM genes that are cancer-related as an estimate of the background frequency of cancer genes in the disease gene population, the probability that 29 genes include 12 cancer-associated genes by chance is 1.4 × 10^-6^. The fraction of OMIM genes that are cancer-related is 0.1 (750 cancer-associated genes in 7381 OMIM genes).

Each of the 8 cancer-related genes listed above plays one or more key roles in processes known to be altered during tumor initiation and development [16]. For example, *MACC1 *is a growth pathway regulator influencing angiogenesis and processes related to metastasis [15]; CDKN2A is a well-studied cell cycle regulator [12] and a known tumor suppressor whose loss results in a diminished ability to regulate growth and predisposition to cancer [11]; ITGB8 has been implicated in activities related to metastasis, including adhesion and migration [14]; and the telomerase enzyme (TERT) is linked to unlimited replication [1]. It is worth noting that CDKN2A, 2B are in strong linkage disequilibrium with rs1412829 at 9p21.3, which has now been identified in 3 independent studies and should therefore be considered an unusually high confidence cancer gene marker.

### Aberrant processes

As noted in the Background, the identification of disease-associated SNPs by GWA studies tends to have low concordance when different populations are compared. An example is the above-mentioned verification in the Mayo Clinic population of only 3 out of the 13 SNPs identified in the AGS population. Our own results identified 12 SNPs based on a meta-analysis of TCGA and AGS. These include 5 of 14 glioma-associated SNPs that had already been verified by at least 2 other studies. There are many reasons for this, including possible differences in ethnicity and other less obvious stratifiers, and differences in sample size and background populations. We believe, however, that an additional reason of an entirely different nature is present which, roughly stated, is that different genes contribute to the same phenotype.

Recent evidence based in clinical epidemiology, computational genomics and various model systems [17-21] suggests that disease phenotypes emerge from dysfunction of one or more components of a functionally coherent gene module. Importantly, the dysfunctions (such as mutations in a gene or its promoter, post-translational modifications, multiple copy number variations, translocations) need not be the same, nor need they be in the same gene, in different individuals with the same disease phenotype. Since alterations in different genes in the same functional module (e.g. a pathway) can lead to the same dysfunction, low reproducibility rates in GWA studies -- aimed at identifying DNA loci having inherited alterations that predispose to complex disorders -- are not surprising. We might, however, expect greater concordance between populations if aberrant functional modules of genes were compared.

In fact we do find that some SNPs not invariant across populations appear in pathways that are invariant, suggesting that the SNPs, however different, are linked to genes that contribute to the same biological process, and that population discordance of SNPs can be partially resolved by evaluating processes rather than genes. Among the 406 AGS SNPs having *p*-value < 0.001, only 3% (12 out of 406) are invariant; i.e. have significant fused *p*-values. This contrasts with 19% of the non-invariant SNPs (75 out of the remaining 394) being in one of the 13 invariant pathways. In addition, as noted above, five genes, each appearing in two or more of these pathways, are invariant across the two populations.

*ITGB8 *was obtained by GWA study and discussed briefly above. Each of the other 4 genes also affects processes involved in cancer progression. More specifically, each has either been previously associated with cancer (*DCC *in the Online Mendelian Inheritance in Man (OMIM) [22]; *FHIT *and *DCC *in the Genome Association Database (GAD) [23]), or belongs to a gene family that has been previously associated with some form of cancer (the Kinase gene *PRKG1*, and the GABA receptor subunit gene *GABRG3*).

(a) *FHIT *(*the fragile histidine triad gene*) is believed to be a tumor suppressor, consistent with its deletion in a number of tumor types [24], including primary brain tumors [25].

(b) The crucial role of dysregulated signalling pathways in cancer development, and the frequent therapeutic targeting of kinases, makes association with the cyclic GMP-dependent protein kinase PRKG1 a plausible finding. This appears to be the first time this particular kinase has been associated with human cancer.

(c) *DCC*, the deleted colorectal carcinoma gene, has (as its name implies) been well studied and has been implicated in a number of cancers in addition to colorectal cancer [26]. *DCC *is an axon guidance receptor that responds to netrin-1 [27], and is a component of a pathway implicated in the regulation of angiogenesis, cell survival, apoptosis, and cell positioning and migration [28], all of which adds to the biological plausibility of this finding.

(d) The GABA-A receptor gene family encodes the major inhibitory neurotransmitter receptors in the central nervous system; changes in GABA-A receptor function have been implicated in diseases as diverse as alcoholism, epilepsy, schizophrenia, autism and Alzheimer's disease. Over 19 different subunit genes code for the pentameric GABA-A receptors, pharmacologically distinct forms of inhibitory neurotransmitter receptors that change their expression levels either during development or in disease [29]. Because of this complexity, elucidating molecular details of its role in many diseases is slow. The ligand GABA may be involved in metastatic prostate cancer [30], making association with aberrant forms of the receptor plausible, and the receptor itself has in fact been implicated in prostate cancer [31], and at least one of its subunits has been found to stimulate the development of pancreatic cancer [32]. This brief context, and the fact that glioma is also a disease of the brain, suggest that the association we find is biologically plausible.

In fact the finding that all 4 genes are plausibly associated with cancer may itself be significant. If we use the conservative estimate that two of the four (*FHIT *and *DCC*) are taken as cancer-associated because of their inclusion in GAD, we obtain 0.0015 as the probability of chance occurrence. We therefore expect that although the procedure we outline is perhaps more heuristic than rigorous, it yields highly suggestive candidates, which should be of interest to glioma researchers.

### Odds ratios

It is evident that the greater the number of risk allele candidates, the greater the chance of identifying a potentially predisposed individual if all such genes are assayed. In addition, however, increasing knowledge of risk alleles affects not just the chance of identification, but the reliability, which will also increase to some extent. The reason for this is related to the chance of observing a combination of risk alleles in the same individual, and the fact that the number of combinations grows as a 2*^r^*, where *r *is the number of known risk alleles. As an example we calculated the odds for all doublet and triplet combinations for the 12 SNPs obtained by a meta-analysis of the AGS and TCGA populations, and list those with odds ratio > 3 in Table S3. Perhaps the most interesting result is the extremely high odds ratio (OR = 5) associated with the two alleles rs1412829 and rs4977**7**56, both of which are in a cyclin dependent kinase.

## Conclusion

In this paper we demonstrate that a meta-analysis of SNP data from TCGA which have not been previously analyzed, together with data from the Adult Glioma Study, identify 12 glioma-associated SNPs which represent 5 genomic regions -- 5p15.33, 9p21.3, 1p21.2, 3q26.2 and 7p15.3 -- three of these not previously reported. Of the 12 SNPs identified in this study, 5 have been previously reported and verified by other studies [1,2], while the remaining 7 are novel candidates. Eight genes known to be cancer-associated are included in, or are in strong linkage disequilibrium with, one or more of these SNPs. An additional 4 genes (*PRKG1, FHIT, GABRG3 *and *DCC *) are identified by a combined pathway enrichment GWA analysis. In all we obtain 29 genes, of which 12 are known to be cancer related.

We have shown that the greatest relative risk occurs when risk alleles are present on chromosomes 1 and 9. Finally based on an analysis of the processes in which candidate genes are involved, we suggest that processes rather than genes are the most informative way to compare different populations, and have shown that such comparisons reduce discordance by approximately 19%.

## Competing interests

The authors declare that they have no competing interests.

## Authors' contributions

TY and CD participated in the design of the study. TY performed the statistical analysis. TY, MK, JH and CD conceived of the study, and participated in its design and coordination and helped to draft the manuscript. All authors read and approved the final manuscript.

## Pre-publication history

The pre-publication history for this paper can be accessed here:

http://www.biomedcentral.com/1755-8794/4/63/prepub

## Supplementary Material

Additional file 1 **Table S1**. We divided the glioma samples from TCGA into 2 independent samples (P1 and P2), conducting a GWA analysis on each using the same i-control population. If the use of a single control created bias, we'd expect overlapping results. In fact the results are quite different. Click here for file

Additional file 2 **Table S2**. The top 406 SNPs reported by Wrensch et al, (2009) with their AGS pvalues (P_1_), TCGA pvalues (P_2_), and Stoufer's combined pvalues(P_12_). Click here for file

Additional file 3 **Table S3**. Pairwise and triplet SNP combinations with odds ratios greater than 3. Combinations of 12 Confirmed Glioma associated SNPs. Click here for file

Additional file 4 **Table S4**. KEGG pathways with SNPs (p < 0.001) from both AGS and TCGA studies. Boldface denotes pathways with significant genes that are common to both populations. Click here for file
