# Supplementary material for: Combinations of newly confirmed Glioma-Associated loci link regions on chromosomes 1 and 9 to increased disease risk
Source: BMC Med Genomics. 2011 Aug 9;4:63. doi: 10.1186/1755-8794-4-63 (PMC3212919; doi:10.1186/1755-8794-4-63)
Supplement: Additional file 1 — Table S1. We divided the glioma samples from TCGA into 2 independent samples (P1 and P2), conducting a GWA analysis on each using the same i-control population. If the use of a single control created bias, we'd expect overlapping results. In fact the results are quite different. [file 1755-8794-4-63-S1.DOC]

Table S1. We divided the glioma samples from TCGA into 2 independent samples (P1 and P2), conducting a GWA analysis on each using the same i-control population. If the use of a single control created bias, we’d expect overlapping results. In fact the results are quite different.

| P1_SNP | P1_PVALUE | P2_SNP | | P2_PVALUE |
| --- | --- | --- | --- | --- |
| rs2160166 | 7.25E-08 | | rs11840214 | 5.57E-08 |
| rs10500337 | 7.92E-08 | | rs1909486 | 2.23E-07 |
| rs8049123 | 8.37E-08 | | rs4772795 | 6.11E-07 |
| rs12910678 | 1.33E-07 | | rs10915621 | 6.29E-07 |
| rs7281472 | 1.63E-07 | | rs13295077 | 9.98E-07 |
| rs10500339 | 3.86E-07 | |  |  |
| rs6557380 | 3.93E-07 | |  |  |
| rs16948970 | 5.42E-07 | |  |  |
| rs17101773 | 7.74E-07 | |  |  |
