# Supplementary material for: Combinations of newly confirmed Glioma-Associated loci link regions on chromosomes 1 and 9 to increased disease risk
Source: BMC Med Genomics. 2011 Aug 9;4:63. doi: 10.1186/1755-8794-4-63 (PMC3212919; doi:10.1186/1755-8794-4-63)
Supplement: Additional file 2 — Table S2. The top 406 SNPs reported by Wrensch et al, (2009) with their AGS pvalues (P1), TCGA pvalues (P2), and Stoufer's combined pvalues(P12). [file 1755-8794-4-63-S2.DOC]

Table S2 The top 406 SNPs reported by Wrensch et al, (2009) with their AGS pvalues (P_1_), TCGA pvalues (P_2_), and Stoufer’s combined pvalues(P_12_).

| SNP | CHR | GENE | FEATURE | AGS (P1) | TCGA(P_2_) | FDR=300,000*N*P_12_ |
| --- | --- | --- | --- | --- | --- | --- |
| **rs2736100** | **5** | **TERT** | **intron** | **5.30E-13** | **2.66E-04** | **7.38E-09** |
| rs11823971 | 11 |  |  | 6.50E-09 | 5.66E-01 | 1.22E+00 |
| **rs1412829** | **9** |  |  | **3.40E-08** | **3.26E-03** | **1.27E-03** |
| rs11163687 | 1 |  |  | 3.60E-08 | 3.13E-01 | 7.41E-01 |
| **rs2157719** | **9** |  |  | **6.10E-08** | **8.00E-03** | **5.40E-03** |
| **rs1063192** | **9** | **CDKN2B** | **UTR-3** | **9.20E-08** | **8.31E-03** | **7.95E-03** |
| rs4809324 | 20 | RTEL1 | intron | 1.50E-07 | 7.66E-01 | 3.06E+01 |
| rs6010620 | 20 | RTEL1 | intron | 1.50E-07 | 1.85E-01 | 7.17E-01 |
| **rs4977756** | **9** |  |  | **4.20E-07** | **1.12E-02** | **3.90E-02** |
| rs10079250 | 5 | CSF1R | missense | 4.40E-07 | 6.38E-01 | 2.69E+01 |
| **rs7530361** | **1** | **SLC35A3** | **intron** | **6.50E-07** | **2.19E-06** | **4.29E-05** |
| **rs501700** | **1** | **HIAT1** | **intron** | **7.10E-07** | **5.99E-06** | **9.72E-05** |
| rs6089953 | 20 | RTEL1 | intron | 7.90E-07 | 2.10E-01 | 2.96E+00 |
| **rs1920116** | **3** | **LRRC31** | **intron** | **1.40E-06** | **2.88E-03** | **2.81E-02** |
| rs1412832 | 9 |  |  | 1.70E-06 | 3.82E-02 | 4.86E-01 |
| **rs506044** | **1** | **CCDC76** | **intron** | **2.10E-06** | **2.45E-06** | **1.57E-04** |
| **rs640030** | **1** | **SASS6** | **intron** | **2.40E-06** | **2.57E-06** | **1.86E-04** |
| **rs687513** | **1** | **CCDC76,SASS6** | **missense,nearGene-5** | **2.90E-06** | **3.91E-06** | **3.03E-04** |
| rs1884043 | 6 |  |  | 2.90E-06 | 6.74E-01 | 1.06E+02 |
| **rs3779505** | **7** | **ITGB8** | **intron** | **3.00E-06** | **5.67E-04** | **1.35E-02** |
| rs12341266 | 9 | RGS3 | intron, missense | 4.80E-06 | 6.16E-03 | 1.70E-01 |
| rs3761034 | 19 | C19orf50 | nearGene-3 | 6.30E-06 | 2.17E-01 | 1.40E+01 |
| rs1333050 | 9 |  |  | 7.40E-06 | 2.01E-01 | 1.39E+01 |
| rs2517552 | 6 | LOC729792 | synon | 9.20E-06 | 5.29E-01 | 1.06E+02 |
| rs1878078 | 2 |  |  | 9.80E-06 | 6.01E-01 | 1.56E+02 |
| rs2844665 | 6 | LOC729792 | intron | 1.10E-05 | 4.23E-01 | 7.05E+01 |
| rs10120688 | 9 |  |  | 1.10E-05 | 3.10E-01 | 3.81E+01 |
| rs12995456 | 2 |  |  | 1.60E-05 | 3.93E-03 | 3.27E-01 |
| rs216148 | 5 | CSF1R | intron | 1.60E-05 | 8.10E-01 | 6.33E+02 |
| rs10936603 | 3 | LOC344657, | intron | 1.90E-05 | 8.12E-03 | 7.35E-01 |
| rs7781339 | 7 |  |  | 1.90E-05 | 7.81E-02 | 7.77E+00 |
| rs2151280 | 9 |  |  | 2.40E-05 | 1.72E-01 | 2.61E+01 |
| rs968698 | 19 |  |  | 2.60E-05 | 1.48E-01 | 2.25E+01 |
| rs2314664 | 19 |  |  | 2.90E-05 | 2.71E-01 | 6.00E+01 |
| rs714447 | 2 |  |  | 3.10E-05 | 9.65E-02 | 1.48E+01 |
| rs10484560 | 6 | C6orf10 | intron | 3.10E-05 | 6.17E-01 | 3.48E+02 |
| rs2301727 | 7 | ITGB8 | intron | 3.20E-05 | 8.27E-02 | 1.26E+01 |
| rs6755968 | 2 |  |  | 3.50E-05 | 7.22E-01 | 6.18E+02 |
| rs219481 | 4 |  |  | 4.20E-05 | 5.46E-01 | 3.06E+02 |
| rs2797634 | 9 |  |  | 4.20E-05 | 3.13E-01 | 9.93E+01 |
| rs2112040 | 2 | SRBD1 | intron | 4.30E-05 | 3.29E-01 | 1.10E+02 |
| rs1377197 | 9 |  |  | 4.60E-05 | 7.06E-01 | 6.75E+02 |
| rs928844 | 21 |  |  | 5.10E-05 | 5.42E-02 | 1.13E+01 |
| rs10116277 | 9 |  |  | 5.30E-05 | 2.12E-01 | 6.36E+01 |
| rs3217992 | 9 | CDKN2B | UTR-3 | 5.40E-05 | 5.65E-01 | 3.90E+02 |
| rs601344 | 1 |  |  | 5.60E-05 | 2.69E-02 | 5.88E+00 |
| rs2839616 | 21 | PKNOX1 | intron | 5.70E-05 | 3.73E-01 | 1.68E+02 |
| rs10182802 | 2 | PARD3B | intron | 5.80E-05 | 7.79E-02 | 1.88E+01 |
| rs2758982 | 10 |  |  | 5.80E-05 | 7.21E-01 | 8.34E+02 |
| rs6066856 | 20 | PREX1 | intron | 5.90E-05 | 5.55E-01 | 3.96E+02 |
| rs843272 | 9 |  |  | 6.10E-05 | 2.60E-01 | 9.57E+01 |
| rs828999 | 1 |  |  | 6.30E-05 | 6.62E-01 | 6.63E+02 |
| rs2074175 | 19 | C19orf60 | cds-reference,missense | 6.30E-05 | 2.87E-01 | 1.15E+02 |
| rs498768 | 12 |  |  | 6.40E-05 | 4.63E-02 | 1.15E+01 |
| rs4809330 | 20 | ZGPAT | intron | 6.70E-05 | 6.28E-01 | 5.94E+02 |
| rs11798 | 7 | AGR3 | UTR-3 | 7.00E-05 | 6.59E-01 | 6.99E+02 |
| rs202147 | 7 | CUTL1 | intron | 7.00E-05 | 5.43E-01 | 4.20E+02 |
| rs9359253 | 6 |  |  | 7.60E-05 | 2.49E-01 | 1.05E+02 |
| rs140040 | 22 |  |  | 7.60E-05 | 7.74E-01 | 1.28E+03 |
| rs1011455 | 15 | GABRG3 | intron | 7.70E-05 | 9.08E-01 | 3.03E+03 |
| rs2490059 | 10 |  |  | 7.80E-05 | 7.19E-01 | 9.87E+02 |
| rs2383207 | 9 |  |  | 8.40E-05 | 4.77E-01 | 3.54E+02 |
| rs10850707 | 12 | TMEM118 | intron | 8.40E-05 | 2.52E-01 | 1.15E+02 |
| rs6457327 | 6 |  |  | 8.60E-05 | 2.05E-01 | 8.64E+01 |
| rs2315008 | 20 | ZGPAT | intron | 8.70E-05 | 5.74E-01 | 5.52E+02 |
| rs4909443 | 8 | COL22A1 | intron | 9.20E-05 | 6.58E-01 | 8.25E+02 |
| rs12021720 | 1 | DBT | cds-reference,missense | 9.40E-05 | 9.74E-06 | 2.27E-02 |
| rs644835 | 1 |  |  | 9.50E-05 | 6.48E-04 | 4.08E-01 |
| rs1790517 | 18 |  |  | 1.00E-04 | 9.17E-01 | 3.75E+03 |
| rs7584568 | 2 | KCNK3 | intron | 0.00011 | 4.42E-01 | 3.63E+02 |
| rs13089423 | 3 |  |  | 0.00011 | 2.45E-01 | 1.34E+02 |
| rs652889 | 3 | PTPRG | intron | 0.00011 | 6.01E-01 | 7.20E+02 |
| rs2517448 | 6 |  |  | 0.00011 | 2.05E-01 | 1.04E+02 |
| rs240919 | 8 |  |  | 0.00011 | 7.89E-01 | 1.71E+03 |
| rs1630231 | 9 |  |  | 0.00011 | 2.87E-02 | 1.11E+01 |
| rs1759039 | 9 |  |  | 0.00011 | 3.68E-01 | 2.58E+02 |
| rs486670 | 13 |  |  | 0.00011 | 2.65E-01 | 1.51E+02 |
| rs7994366 | 13 |  |  | 0.00011 | 1.03E-01 | 4.32E+01 |
| rs1345442 | 16 | LITAF,LOC729963 | intron,nearGene-3 | 0.00011 | 8.50E-02 | 3.45E+01 |
| rs2847197 | 18 |  |  | 0.00011 | 9.17E-01 | 3.93E+03 |
| rs12990755 | 2 |  |  | 0.00012 | 7.36E-01 | 1.38E+03 |
| rs2060390 | 2 |  |  | 0.00012 | 4.84E-02 | 2.02E+01 |
| rs12367885 | 12 |  |  | 0.00012 | 9.03E-01 | 3.66E+03 |
| rs10494090 | 1 | SLC25A24 | intron | 0.00013 | 3.11E-01 | 2.19E+02 |
| rs2810424 | 1 | DBT | intron | 0.00013 | 1.10E-05 | 3.51E-02 |
| rs1514221 | 8 |  |  | 0.00013 | 2.07E-01 | 1.19E+02 |
| rs7304729 | 12 |  |  | 0.00013 | 1.95E-01 | 1.10E+02 |
| rs4981369 | 14 |  |  | 0.00013 | 9.04E-01 | 3.87E+03 |
| rs3746816 | 20 | PREX1 | intron | 0.00013 | 2.30E-01 | 1.38E+02 |
| rs2307068 | 3 |  |  | 0.00014 | 6.51E-01 | 1.04E+03 |
| rs9819059 | 3 |  |  | 0.00014 | 6.55E-01 | 1.06E+03 |
| rs2287197 | 16 | HEATR3 | cds-synon,cds-reference | 0.00014 | 2.37E-01 | 1.52E+02 |
| rs1560691 | 19 | NLRP5 | cds-reference,cds-synon | 0.00014 | 5.48E-03 | 3.15E+00 |
| rs6701658 | 1 |  |  | 0.00015 | 3.36E-04 | 4.02E-01 |
| rs6844588 | 4 | MLF1IP | intron | 0.00015 | 3.73E-01 | 3.27E+02 |
| rs6597439 | 7 | DPP6 | intron | 0.00015 | 1.62E-01 | 9.57E+01 |
| rs2579776 | 10 |  |  | 0.00015 | 2.47E-01 | 1.70E+02 |
| rs8051216 | 16 | HEATR3 | intron | 0.00015 | 2.57E-01 | 1.81E+02 |
| rs877639 | 16 | HEATR3 | intron | 0.00015 | 3.79E-01 | 3.36E+02 |
| rs2175604 | 17 |  |  | 0.00016 | 5.77E-01 | 8.25E+02 |
| rs2803396 | 1 |  |  | 0.00017 | 7.65E-01 | 1.95E+03 |
| rs6552804 | 4 | MLF1IP | missense,cds-reference | 0.00017 | 3.79E-01 | 3.66E+02 |
| rs3807936 | 7 | ITGB8 | intron | 0.00017 | 4.13E-03 | 2.99E+00 |
| rs12115052 | 8 |  |  | 0.00017 | 2.52E-01 | 1.92E+02 |
| rs1871457 | 14 |  |  | 0.00017 | 2.08E-01 | 1.46E+02 |
| rs879471 | 20 | STMN3 | nearGene-3 | 0.00017 | 6.20E-01 | 1.03E+03 |
| rs11166389 | 1 |  |  | 0.00018 | 6.68E-04 | 7.86E-01 |
| rs3995732 | 6 |  |  | 0.00018 | 9.80E-01 | 1.16E+04 |
| rs2485901 | 1 |  |  | 0.00019 | 9.54E-01 | 7.68E+03 |
| rs6725882 | 2 |  |  | 2.00E-04 | 8.33E-01 | 3.06E+03 |
| rs1495908 | 3 |  |  | 2.00E-04 | 6.55E-01 | 1.32E+03 |
| rs9289589 | 3 |  |  | 2.00E-04 | 4.83E-01 | 6.45E+02 |
| rs877276 | 4 | MLF1IP | intron | 2.00E-04 | 3.73E-01 | 3.99E+02 |
| rs1047064 | 7 |  |  | 2.00E-04 | 8.03E-01 | 2.60E+03 |
| rs1379072 | 5 |  |  | 0.00021 | 8.09E-01 | 2.75E+03 |
| rs10966423 | 9 |  |  | 0.00021 | 1.09E-01 | 7.68E+01 |
| rs4751828 | 10 |  |  | 0.00021 | 7.90E-01 | 2.50E+03 |
| rs10501352 | 11 |  |  | 0.00021 | 9.36E-01 | 6.57E+03 |
| rs2211938 | 21 |  |  | 0.00021 | 2.83E-02 | 1.91E+01 |
| rs4132013 | 7 | LHFPL3 | intron | 0.00022 | 9.74E-01 | 1.12E+04 |
| rs985598 | 11 |  |  | 0.00022 | 7.61E-01 | 2.23E+03 |
| rs11107628 | 12 |  |  | 0.00022 | 8.15E-01 | 2.92E+03 |
| rs328994 | 18 | PPP4R1 | intron | 0.00022 | 8.66E-02 | 6.15E+01 |
| rs4400745 | 10 | PRKG1 | intron | 0.00023 | 6.33E-01 | 1.31E+03 |
| rs3755527 | 2 |  |  | 0.00024 | 4.14E-01 | 5.46E+02 |
| rs2659690 | 3 | DJB8 | cds-synon,cds-reference | 0.00024 | 4.65E-01 | 6.78E+02 |
| rs8181298 | 10 |  |  | 0.00025 | 5.79E-01 | 1.11E+03 |
| rs2158250 | 7 | ITGB8 | intron | 0.00026 | 1.52E-01 | 1.35E+02 |
| rs10115162 | 9 |  |  | 0.00026 | 5.41E-01 | 9.75E+02 |
| rs12766217 | 10 |  |  | 0.00026 | 8.69E-01 | 4.38E+03 |
| rs17026260 | 12 |  |  | 0.00026 | 4.65E-01 | 7.14E+02 |
| rs7922737 | 10 |  |  | 0.00027 | 4.03E-01 | 5.64E+02 |
| rs4074052 | 8 |  |  | 0.00028 | 3.48E-01 | 4.50E+02 |
| rs485219 | 8 |  |  | 0.00028 | 3.39E-01 | 4.32E+02 |
| rs10506662 | 12 |  |  | 0.00028 | 8.16E-02 | 6.99E+01 |
| rs7153013 | 14 |  |  | 0.00028 | 9.68E-01 | 1.13E+04 |
| rs3826301 | 17 | MSI2 | intron,UTR-3 | 0.00028 | 1.18E-01 | 1.06E+02 |
| rs11203567 | 8 |  |  | 0.00029 | 5.69E-01 | 1.17E+03 |
| rs10507337 | 13 |  |  | 0.00029 | 1.46E-01 | 1.40E+02 |
| rs7145814 | 14 | OR5AU1 | cds-reference,missense | 0.00029 | 7.14E-01 | 2.12E+03 |
| rs1862802 | 16 |  |  | 0.00029 | 5.43E-01 | 1.06E+03 |
| rs12712184 | 2 |  |  | 3.00E-04 | 4.72E-01 | 8.10E+02 |
| rs4665352 | 2 |  |  | 3.00E-04 | 2.69E-01 | 3.18E+02 |
| rs4522850 | 4 |  |  | 3.00E-04 | 2.39E-01 | 2.70E+02 |
| rs6818704 | 4 |  |  | 3.00E-04 | 6.10E-01 | 1.41E+03 |
| rs6882023 | 5 |  |  | 3.00E-04 | 1.02E-01 | 9.45E+01 |
| rs10784000 | 12 |  |  | 3.00E-04 | 8.43E-01 | 4.05E+03 |
| rs2615100 | 8 |  |  | 0.00031 | 2.45E-01 | 2.87E+02 |
| rs6597745 | 10 | ADAM12 | intron | 0.00031 | 4.68E-01 | 8.13E+02 |
| rs4889242 | 16 | PKD1L2 | intron | 0.00031 | 8.40E-01 | 4.05E+03 |
| rs6840028 | 4 | LOC729407 | intron | 0.00032 | 5.23E-01 | 1.04E+03 |
| rs4719617 | 7 |  |  | 0.00032 | 4.51E-01 | 7.74E+02 |
| rs4466778 | 10 | PRKG1 | intron | 0.00032 | 6.63E-01 | 1.82E+03 |
| rs10774021 | 12 | SLC6A13 | intron | 0.00032 | 5.50E-01 | 1.16E+03 |
| rs1601717 | 20 |  |  | 0.00032 | 1.26E-01 | 1.26E+02 |
| rs6797956 | 3 |  |  | 0.00033 | 3.42E-01 | 4.92E+02 |
| rs1265086 | 6 | CCHCR1 | nearGene-3 | 0.00033 | 4.00E-01 | 6.39E+02 |
| rs1699101 | 11 |  |  | 0.00033 | 2.87E-01 | 3.75E+02 |
| rs8007901 | 14 | C14orf70 | intron | 0.00033 | 8.42E-01 | 4.23E+03 |
| rs7529576 | 1 |  |  | 0.00034 | 7.27E-01 | 2.47E+03 |
| rs12114224 | 8 | MMP16 | intron | 0.00034 | 6.20E-01 | 1.59E+03 |
| rs1531136 | 3 |  |  | 0.00035 | 2.82E-02 | 2.96E+01 |
| rs1344307 | 7 |  |  | 0.00035 | 2.63E-01 | 3.45E+02 |
| rs4721701 | 7 |  |  | 0.00035 | 6.16E-01 | 1.59E+03 |
| rs3741061 | 11 |  |  | 0.00035 | 8.61E-01 | 4.89E+03 |
| rs4791036 | 17 |  |  | 0.00035 | 7.05E-02 | 7.20E+01 |
| rs2830538 | 21 |  |  | 0.00035 | 4.90E-01 | 9.66E+02 |
| rs3130501 | 6 | POU5F1 | intron | 0.00036 | 8.15E-01 | 3.87E+03 |
| rs3130559 | 6 |  |  | 0.00036 | 2.32E-01 | 2.97E+02 |
| rs232291 | 20 | WFDC13 | intron | 0.00036 | 3.39E-01 | 5.16E+02 |
| rs6587440 | 1 |  |  | 0.00037 | 4.20E-01 | 7.50E+02 |
| rs10496014 | 2 |  |  | 0.00037 | 4.96E-01 | 1.03E+03 |
| rs9832635 | 3 |  |  | 0.00037 | 2.33E-02 | 2.61E+01 |
| rs4135165 | 9 |  |  | 0.00037 | 5.03E-01 | 1.05E+03 |
| rs9933173 | 16 |  |  | 0.00038 | 9.08E-01 | 6.99E+03 |
| rs2350051 | 4 |  |  | 0.00039 | 1.58E-01 | 1.93E+02 |
| rs6828619 | 4 |  |  | 0.00039 | 8.52E-01 | 4.92E+03 |
| rs208346 | 7 |  |  | 0.00039 | 8.94E-01 | 6.42E+03 |
| rs540029 | 11 |  |  | 0.00039 | 2.25E-01 | 3.03E+02 |
| rs12125049 | 1 |  |  | 4.00E-04 | 4.36E-01 | 8.46E+02 |
| rs4600036 | 1 | RLF | intron | 4.00E-04 | 3.37E-01 | 5.52E+02 |
| rs6662846 | 1 | CRYZ | nearGene-3 | 4.00E-04 | 4.83E-01 | 1.03E+03 |
| rs10058297 | 5 |  |  | 4.00E-04 | 1.35E-01 | 1.63E+02 |
| rs4012480 | 9 |  |  | 4.00E-04 | 5.55E-01 | 1.36E+03 |
| rs1999294 | 21 |  |  | 4.00E-04 | 7.58E-02 | 8.64E+01 |
| rs1603867 | 3 |  |  | 0.00041 | 9.50E-01 | 1.07E+04 |
| rs10489024 | 4 |  |  | 0.00041 | 1.37E-01 | 1.69E+02 |
| rs1386689 | 8 | PSD3 | intron | 0.00041 | 1.45E-01 | 1.81E+02 |
| rs2826903 | 21 |  |  | 0.00041 | 6.72E-01 | 2.20E+03 |
| rs4309013 | 1 |  |  | 0.00042 | 3.75E-01 | 6.75E+02 |
| rs3864070 | 3 |  |  | 0.00042 | 6.09E-01 | 1.74E+03 |
| rs4367471 | 7 | LHFPL3 | intron | 0.00042 | 5.17E-01 | 1.21E+03 |
| rs2638648 | 8 | PSD3 | intron | 0.00042 | 2.28E-01 | 3.24E+02 |
| rs11669114 | 19 |  |  | 0.00042 | 7.47E-01 | 3.06E+03 |
| rs6700346 | 1 |  |  | 0.00043 | 2.56E-01 | 3.87E+02 |
| rs1405940 | 4 | CCDC111 | intron | 0.00043 | 1.55E-01 | 2.03E+02 |
| rs3734960 | 7 |  |  | 0.00043 | 5.16E-01 | 1.23E+03 |
| rs6465403 | 7 |  |  | 0.00043 | 4.85E-02 | 5.88E+01 |
| rs3829072 | 9 | SMARCA2 | intron | 0.00043 | 3.99E-01 | 7.62E+02 |
| rs4238897 | 16 |  |  | 0.00043 | 8.97E-01 | 6.90E+03 |
| rs7861396 | 9 | JMJD2C | intron | 0.00044 | 4.05E-01 | 7.95E+02 |
| rs2165406 | 14 |  |  | 0.00044 | 4.07E-01 | 8.01E+02 |
| rs2425398 | 20 |  |  | 0.00044 | 5.19E-01 | 1.26E+03 |
| rs6897488 | 5 | GRAMD3 | intron | 0.00045 | 3.92E-01 | 7.62E+02 |
| rs7929741 | 11 |  |  | 0.00045 | 7.11E-01 | 2.73E+03 |
| rs8001568 | 13 |  |  | 0.00045 | 3.11E-01 | 5.31E+02 |
| rs1297111 | 15 |  |  | 0.00045 | 8.35E-01 | 4.86E+03 |
| rs2306134 | 18 | PPP4R1 | missense,cds-reference | 0.00045 | 5.63E-02 | 7.05E+01 |
| rs9966123 | 18 |  |  | 0.00045 | 4.49E-01 | 9.66E+02 |
| rs190963 | 1 |  |  | 0.00046 | 5.61E-02 | 7.17E+01 |
| rs4654103 | 1 | SMYD3 | intron | 0.00046 | 1.60E-02 | 2.28E+01 |
| rs11708509 | 3 |  |  | 0.00046 | 1.09E-01 | 1.43E+02 |
| rs868691 | 10 |  |  | 0.00046 | 9.06E-01 | 7.62E+03 |
| rs12088062 | 1 |  |  | 0.00047 | 2.09E-01 | 3.15E+02 |
| rs7555310 | 1 | OR2M7 | missense,cds-reference | 0.00047 | 4.31E-01 | 9.27E+02 |
| rs11729739 | 4 |  |  | 0.00047 | 9.02E-01 | 7.47E+03 |
| rs17497950 | 12 |  |  | 0.00047 | 6.09E-01 | 1.87E+03 |
| rs11679767 | 2 |  |  | 0.00048 | 1.13E-02 | 1.77E+01 |
| rs7603271 | 2 | SPP2 | intron | 0.00048 | 8.49E-01 | 5.43E+03 |
| rs4642918 | 1 |  |  | 0.00049 | 3.56E-01 | 6.93E+02 |
| rs4658619 | 1 | PLD5 | intron | 0.00049 | 3.88E-01 | 7.95E+02 |
| rs1789590 | 18 |  |  | 0.00049 | 7.16E-01 | 2.94E+03 |
| rs2985441 | 1 | LOC730144 | missense,cds-reference | 5.00E-04 | 3.89E-01 | 8.10E+02 |
| rs745299 | 15 |  |  | 5.00E-04 | 2.50E-01 | 4.20E+02 |
| rs4823719 | 22 |  |  | 5.00E-04 | 9.09E-01 | 8.13E+03 |
| rs323716 | 1 | CCDC23 | intron | 0.00051 | 8.33E-02 | 1.16E+02 |
| rs706041 | 7 |  |  | 0.00051 | 3.56E-03 | 7.53E+00 |
| rs7189692 | 16 | LITAF | intron | 0.00051 | 5.07E-01 | 1.32E+03 |
| rs6084875 | 20 |  |  | 0.00051 | 5.67E-01 | 1.67E+03 |
| rs1178340 | 7 |  |  | 0.00052 | 7.94E-01 | 4.29E+03 |
| rs1426601 | 8 |  |  | 0.00052 | 1.29E-01 | 1.90E+02 |
| rs6500818 | 16 | A2BP1 | intron | 0.00052 | 7.11E-01 | 2.98E+03 |
| rs4655454 | 1 |  |  | 0.00053 | 1.91E-02 | 3.00E+01 |
| rs1028285 | 22 |  |  | 0.00053 | 7.38E-02 | 1.06E+02 |
| rs1409785 | 1 |  |  | 0.00054 | 1.57E-01 | 2.46E+02 |
| rs655146 | 1 |  |  | 0.00054 | 5.72E-01 | 1.77E+03 |
| rs6712180 | 2 |  |  | 0.00054 | 8.49E-01 | 5.79E+03 |
| rs4877005 | 9 |  |  | 0.00054 | 1.88E-01 | 3.06E+02 |
| rs10778367 | 12 | ALDH1L2 | intron | 0.00054 | 8.47E-01 | 5.73E+03 |
| rs1974821 | 19 | SIGLEC10 | cds-reference,cds-synon | 0.00054 | 8.39E-01 | 5.49E+03 |
| rs9974272 | 21 |  |  | 0.00054 | 2.67E-01 | 4.86E+02 |
| rs17110757 | 1 | ACOT11 | intron | 0.00055 | 3.57E-01 | 7.53E+02 |
| rs12515890 | 5 |  |  | 0.00055 | 1.36E-01 | 2.11E+02 |
| rs10517336 | 4 |  |  | 0.00056 | 6.26E-01 | 2.22E+03 |
| rs39183 | 7 | THSD7A | intron | 0.00056 | 1.61E-01 | 2.61E+02 |
| rs2024366 | 7 |  |  | 0.00057 | 1.30E-01 | 2.06E+02 |
| rs2294938 | 1 | LOC642587 | intron | 0.00058 | 6.25E-01 | 2.27E+03 |
| rs4666020 | 2 | RBKS | intron | 0.00058 | 3.54E-01 | 7.74E+02 |
| rs4973316 | 2 | LOC93349 | intron | 0.00058 | 8.60E-01 | 6.42E+03 |
| rs7613341 | 3 |  |  | 0.00058 | 6.73E-01 | 2.73E+03 |
| rs795998 | 4 |  |  | 0.00058 | 8.54E-01 | 6.21E+03 |
| rs12538781 | 7 |  |  | 0.00058 | 9.51E-01 | 1.28E+04 |
| rs834822 | 7 |  |  | 0.00058 | 8.40E-01 | 5.73E+03 |
| rs1333040 | 9 |  |  | 0.00058 | 3.44E-01 | 7.38E+02 |
| rs6495246 | 15 |  |  | 0.00058 | 3.85E-01 | 8.85E+02 |
| rs1043149 | 17 |  |  | 0.00058 | 9.33E-03 | 1.80E+01 |
| rs2837990 | 21 |  |  | 0.00058 | 5.80E-01 | 1.91E+03 |
| rs10492371 | 12 | PPFIBP1 | intron | 6.00E-04 | 8.63E-01 | 6.63E+03 |
| rs9430161 | 1 |  |  | 0.00061 | 1.12E-01 | 1.85E+02 |
| rs1554668 | 4 |  |  | 0.00061 | 7.28E-01 | 3.51E+03 |
| rs1417032 | 10 |  |  | 0.00061 | 4.98E-01 | 1.44E+03 |
| rs6497081 | 15 |  |  | 0.00061 | 5.35E-01 | 1.66E+03 |
| rs8100291 | 19 |  |  | 0.00061 | 2.15E-01 | 3.99E+02 |
| rs1001628 | 2 |  |  | 0.00062 | 3.24E-01 | 7.08E+02 |
| rs7647589 | 3 | LRRC31 | intron | 0.00062 | 2.25E-01 | 4.26E+02 |
| rs1451904 | 12 | TMEM132D | intron | 0.00062 | 7.13E-01 | 3.33E+03 |
| rs2155945 | 18 |  |  | 0.00062 | 8.11E-02 | 1.33E+02 |
| rs12668172 | 7 |  |  | 0.00063 | 9.89E-01 | 2.58E+04 |
| rs748454 | 20 |  |  | 0.00063 | 1.28E-02 | 2.52E+01 |
| rs1992372 | 14 |  |  | 0.00064 | 9.63E-01 | 1.55E+04 |
| rs1028286 | 22 |  |  | 0.00064 | 7.39E-02 | 1.24E+02 |
| rs10484796 | 6 |  |  | 0.00065 | 6.94E-01 | 3.18E+03 |
| rs6937502 | 6 |  |  | 0.00065 | 6.31E-01 | 2.49E+03 |
| rs4955628 | 3 |  |  | 0.00066 | 3.94E-01 | 1.01E+03 |
| rs7330519 | 13 |  |  | 0.00066 | 7.71E-02 | 1.33E+02 |
| rs9304040 | 18 | PPP4R1 | intron | 0.00066 | 3.60E-02 | 6.39E+01 |
| rs1770566 | 1 |  |  | 0.00067 | 5.02E-01 | 1.56E+03 |
| rs7617530 | 3 | FHIT | intron | 0.00067 | 2.52E-02 | 4.71E+01 |
| rs17475118 | 5 |  |  | 0.00067 | 2.00E-01 | 3.90E+02 |
| rs2815727 | 6 |  |  | 0.00067 | 3.22E-02 | 5.88E+01 |
| rs2240601 | 17 |  |  | 0.00067 | 8.95E-01 | 8.58E+03 |
| rs4793867 | 17 | MSI2 | intron | 0.00067 | 3.59E-01 | 8.73E+02 |
| rs2208123 | 22 |  |  | 0.00067 | 5.35E-01 | 1.76E+03 |
| rs4386305 | 2 |  |  | 0.00068 | 7.97E-01 | 5.07E+03 |
| rs4851692 | 2 |  |  | 0.00069 | 2.90E-01 | 6.51E+02 |
| rs6885628 | 5 |  |  | 0.00069 | 6.91E-01 | 3.27E+03 |
| rs6465408 | 7 |  |  | 0.00069 | 6.48E-01 | 2.76E+03 |
| rs728166 | 12 |  |  | 0.00069 | 1.52E-01 | 2.87E+02 |
| rs8094970 | 18 |  |  | 0.00069 | 3.31E-01 | 7.89E+02 |
| rs140522 | 22 |  |  | 0.00069 | 3.47E-01 | 8.46E+02 |
| rs10510207 | 3 | CNTN6 | intron | 7.00E-04 | 8.83E-01 | 8.13E+03 |
| rs761339 | 6 |  |  | 7.00E-04 | 4.47E-01 | 1.30E+03 |
| rs604000 | 13 |  |  | 7.00E-04 | 8.03E-01 | 5.31E+03 |
| rs7574757 | 2 |  |  | 0.00072 | 3.44E-01 | 8.61E+02 |
| rs12663163 | 6 |  |  | 0.00072 | 3.11E-01 | 7.41E+02 |
| rs2504897 | 6 |  |  | 0.00072 | 2.58E-02 | 5.13E+01 |
| rs7904336 | 10 |  |  | 0.00072 | 9.06E-01 | 9.63E+03 |
| rs13323146 | 3 |  |  | 0.00073 | 7.06E-01 | 3.57E+03 |
| rs2454585 | 5 | ITGA1 | intron | 0.00073 | 6.62E-01 | 3.00E+03 |
| rs10509857 | 10 |  |  | 0.00073 | 7.08E-01 | 3.60E+03 |
| rs11605303 | 11 |  |  | 0.00073 | 1.81E-02 | 3.84E+01 |
| rs1876527 | 6 |  |  | 0.00074 | 7.08E-01 | 3.63E+03 |
| rs2277084 | 6 | LAMA4 | intron | 0.00074 | 1.89E-02 | 4.02E+01 |
| rs10491833 | 9 |  |  | 0.00074 | 3.87E-01 | 1.06E+03 |
| rs7041637 | 9 |  |  | 0.00074 | 6.23E-01 | 2.62E+03 |
| rs7975313 | 12 |  |  | 0.00074 | 1.34E-01 | 2.62E+02 |
| rs6022123 | 20 |  |  | 0.00074 | 2.47E-01 | 5.49E+02 |
| rs6084884 | 20 |  |  | 0.00074 | 8.11E-01 | 5.70E+03 |
| rs11701851 | 21 |  |  | 0.00074 | 3.20E-01 | 7.89E+02 |
| rs10924303 | 1 | KIF26B | intron | 0.00075 | 6.95E-01 | 3.48E+03 |
| rs6600320 | 1 | RLF | intron | 0.00075 | 2.92E-01 | 6.99E+02 |
| rs1401504 | 2 | LOC727890 | cds-reference,missense | 0.00075 | 7.24E-01 | 3.93E+03 |
| rs10516497 | 4 |  |  | 0.00076 | 5.62E-01 | 2.12E+03 |
| rs443277 | 5 |  |  | 0.00076 | 6.82E-01 | 3.33E+03 |
| rs11615170 | 12 | PTPRB | intron | 0.00076 | 1.38E-01 | 2.77E+02 |
| rs7313849 | 12 | B4GALNT3 | intron | 0.00076 | 8.74E-01 | 8.04E+03 |
| rs714975 | 14 | RGS6 | intron | 0.00076 | 3.27E-01 | 8.31E+02 |
| rs4887546 | 15 |  |  | 0.00076 | 1.21E-01 | 2.39E+02 |
| rs10871565 | 18 |  |  | 0.00076 | 4.08E-01 | 1.17E+03 |
| rs2267903 | 2 | SPP2 | intron | 0.00077 | 8.17E-01 | 6.00E+03 |
| rs10936294 | 3 |  |  | 0.00077 | 1.74E-01 | 3.66E+02 |
| rs9451486 | 6 |  |  | 0.00077 | 8.07E-02 | 1.58E+02 |
| rs870168 | 2 |  |  | 0.00078 | 4.90E-01 | 1.64E+03 |
| rs7635168 | 3 |  |  | 0.00078 | 4.90E-01 | 1.64E+03 |
| rs6899965 | 6 |  |  | 0.00078 | 7.66E-01 | 4.77E+03 |
| rs615545 | 7 |  |  | 0.00078 | 2.79E-01 | 6.75E+02 |
| rs673377 | 11 |  |  | 0.00078 | 1.23E-01 | 2.49E+02 |
| rs8058333 | 16 |  |  | 0.00078 | 2.11E-01 | 4.68E+02 |
| rs9353399 | 6 |  |  | 0.00079 | 2.64E-01 | 6.30E+02 |
| rs10496905 | 2 | LRP1B | intron | 8.00E-04 | 3.68E-01 | 1.03E+03 |
| rs288193 | 5 |  |  | 8.00E-04 | 9.74E-01 | 2.03E+04 |
| rs7711912 | 5 |  |  | 8.00E-04 | 3.25E-01 | 8.52E+02 |
| rs525843 | 9 |  |  | 8.00E-04 | 8.74E-01 | 8.28E+03 |
| rs1011456 | 15 |  |  | 8.00E-04 | 8.82E-01 | 8.67E+03 |
| rs3914576 | 15 |  |  | 8.00E-04 | 6.94E-02 | 1.40E+02 |
| rs1435372 | 3 |  |  | 0.00081 | 4.96E-01 | 1.73E+03 |
| rs8059726 | 16 |  |  | 0.00081 | 3.97E-01 | 1.17E+03 |
| rs11583706 | 1 |  |  | 0.00082 | 8.97E-01 | 9.69E+03 |
| rs2053918 | 2 | VWA3B | intron | 0.00082 | 2.76E-02 | 6.12E+01 |
| rs4427071 | 7 |  |  | 0.00082 | 2.57E-01 | 6.27E+02 |
| rs11265889 | 9 |  |  | 0.00082 | 2.30E-02 | 5.22E+01 |
| rs589248 | 9 |  |  | 0.00082 | 7.16E-02 | 1.48E+02 |
| rs755492 | 14 |  |  | 0.00082 | 7.32E-01 | 4.26E+03 |
| rs8004291 | 14 |  |  | 0.00082 | 6.79E-01 | 3.45E+03 |
| rs7073924 | 10 | SORCS1 | intron | 0.00083 | 7.62E-01 | 4.86E+03 |
| rs16838813 | 1 |  |  | 0.00085 | 9.09E-02 | 1.94E+02 |
| rs1899025 | 2 |  |  | 0.00085 | 1.94E-02 | 4.68E+01 |
| rs1860293 | 4 |  |  | 0.00085 | 9.92E-01 | 3.27E+04 |
| rs42077 | 7 |  |  | 0.00085 | 1.92E-01 | 4.44E+02 |
| rs639813 | 10 | VWA2 | intron | 0.00085 | 4.85E-01 | 1.71E+03 |
| rs7153709 | 14 |  |  | 0.00085 | 2.80E-02 | 6.39E+01 |
| rs1980444 | 1 | PLA2G4A | intron | 0.00086 | 5.73E-01 | 2.39E+03 |
| rs17008912 | 4 |  |  | 0.00086 | 8.45E-01 | 7.32E+03 |
| rs202159 | 7 | CUTL1 | intron | 0.00086 | 4.81E-01 | 1.70E+03 |
| rs11200146 | 10 | ATE1 | intron | 0.00086 | 6.32E-01 | 2.98E+03 |
| rs805674 | 10 |  |  | 0.00086 | 2.95E-01 | 7.83E+02 |
| rs1372796 | 11 |  |  | 0.00086 | 8.35E-01 | 6.96E+03 |
| rs4968515 | 17 |  |  | 0.00086 | 9.84E-01 | 2.55E+04 |
| rs2517532 | 6 |  |  | 0.00087 | 3.93E-01 | 1.21E+03 |
| rs2874620 | 13 |  |  | 0.00087 | 1.46E-01 | 3.27E+02 |
| rs4923889 | 15 |  |  | 0.00087 | 2.34E-01 | 5.79E+02 |
| rs87061 | 1 |  |  | 0.00088 | 4.31E-02 | 9.69E+01 |
| rs9436297 | 1 |  |  | 0.00088 | 9.16E-01 | 1.15E+04 |
| rs3911081 | 2 |  |  | 0.00088 | 1.11E-02 | 3.06E+01 |
| rs6454657 | 6 |  |  | 0.00088 | 4.89E-01 | 1.78E+03 |
| rs6962818 | 7 |  |  | 0.00088 | 4.92E-01 | 1.79E+03 |
| rs800583 | 8 |  |  | 0.00088 | 5.36E-01 | 2.12E+03 |
| rs10924690 | 1 | SMYD3 | intron | 0.00089 | 3.76E-01 | 1.15E+03 |
| rs16829231 | 2 |  |  | 0.00089 | 9.53E-01 | 1.61E+04 |
| rs2172347 | 3 |  |  | 0.00089 | 9.65E-01 | 1.85E+04 |
| rs4734508 | 8 |  |  | 0.00089 | 8.63E-01 | 8.22E+03 |
| rs11854054 | 15 |  |  | 0.00089 | 2.59E-01 | 6.72E+02 |
| rs12522240 | 5 |  |  | 9.00E-04 | 8.49E-01 | 7.68E+03 |
| rs9585170 | 13 |  |  | 9.00E-04 | 2.49E-01 | 6.42E+02 |
| rs3001156 | 1 |  |  | 0.00091 | 3.91E-02 | 9.12E+01 |
| rs1868110 | 3 |  |  | 0.00091 | 2.46E-01 | 6.39E+02 |
| rs11244887 | 10 | ADAM12 | intron | 0.00091 | 1.17E-01 | 2.66E+02 |
| rs4833346 | 4 |  |  | 0.00092 | 2.91E-01 | 8.07E+02 |
| rs4815763 | 20 |  |  | 0.00092 | 9.79E-01 | 2.36E+04 |
| rs3218020 | 9 |  |  | 0.00093 | 8.65E-01 | 8.52E+03 |
| rs12602254 | 17 |  |  | 0.00093 | 7.49E-01 | 4.92E+03 |
| rs2027372 | 1 | SMYD3 | intron | 0.00094 | 5.17E-03 | 1.80E+01 |
| rs11729868 | 4 |  |  | 0.00094 | 1.00E+00 | 1.93E+03 |
| rs673127 | 12 |  |  | 0.00094 | 1.99E-01 | 5.01E+02 |
| rs7971858 | 12 |  |  | 0.00094 | 1.27E-01 | 2.99E+02 |
| rs6466757 | 7 | KCND2 | intron | 0.00095 | 8.05E-01 | 6.36E+03 |
| rs4774257 | 15 |  |  | 0.00095 | 1.79E-01 | 4.44E+02 |
| rs3751859 | 16 |  |  | 0.00095 | 2.38E-01 | 6.30E+02 |
| rs4794519 | 17 |  |  | 0.00095 | 1.46E-01 | 3.51E+02 |
| rs1056408 | 18 |  |  | 0.00095 | 7.22E-01 | 4.47E+03 |
| rs1056294 | 19 |  |  | 0.00095 | 1.26E-01 | 2.99E+02 |
| rs12723208 | 1 | TRAF5 | intron | 0.00096 | 6.89E-01 | 3.96E+03 |
| rs855679 | 7 | SSPO | intron | 0.00096 | 3.48E-01 | 1.07E+03 |
| rs2849492 | 18 | DT | intron | 0.00096 | 6.82E-01 | 3.84E+03 |
| rs7591929 | 2 |  |  | 0.00097 | 1.94E-02 | 5.25E+01 |
| rs970456 | 5 |  |  | 0.00097 | 3.85E-01 | 1.26E+03 |
| rs1922617 | 16 |  |  | 0.00097 | 4.16E-01 | 1.43E+03 |
| rs1145245 | 18 |  |  | 0.00098 | 6.97E-01 | 4.14E+03 |
| rs2353550 | 1 |  |  | 0.00099 | 4.02E-01 | 1.37E+03 |
| rs1986361 | 9 |  |  | 0.00099 | 8.13E-01 | 6.78E+03 |
| rs2461682 | 11 |  |  | 0.00099 | 1.03E-01 | 2.49E+02 |
| rs8114703 | 20 |  |  | 0.00099 | 7.09E-01 | 4.35E+03 |
| rs6743450 | 2 |  |  | 0.001 | 1.21E-01 | 2.98E+02 |
| rs3740469 | 10 | SLK | cds-reference,missense | 0.001 | 3.88E-01 | 1.31E+03 |
| rs765651 | 10 |  |  | 0.001 | 3.43E-01 | 1.08E+03 |
| rs9585777 | 13 |  |  | 0.001 | 2.57E-01 | 7.26E+02 |
