# Supplementary material for: Combinations of newly confirmed Glioma-Associated loci link regions on chromosomes 1 and 9 to increased disease risk
Source: BMC Med Genomics. 2011 Aug 9;4:63. doi: 10.1186/1755-8794-4-63 (PMC3212919; doi:10.1186/1755-8794-4-63)
Supplement: Additional file 3 — Table S3. Pairwise and triplet SNP combinations with odds ratios greater than 3. Combinations of 12 Confirmed Glioma associated SNPs. [file 1755-8794-4-63-S3.DOC]

Table S3. Pairwise and triplet SNP combinations with odds ratios greater than 3. Combinations of 12 Confirmed Glioma associated SNPs.

| SNP Combinations | | | RISK ALLELE | Freq | OR | Pvalue_OR_ |
| --- | --- | --- | --- | --- | --- | --- |
| *rs1412829 | | *rs4977756 | 12 | 7.07E-02 | 5.04 | 8.55E-31 |
| *rs1412829 | | #rs7530361 | 11 | 5.45E-02 | 3.31 | 3.58E-07 |
| *rs1412829 | | #rs501700 | 11 | 5.51E-02 | 3.09 | 1.95E-06 |
| *rs1412829 | | #rs506044 | 11 | 5.47E-02 | 3.23 | 5.15E-07 |
| *rs1412829 | | #rs640030 | 11 | 5.42E-02 | 3.28 | 4.30E-07 |
| *rs1412829 | | #rs687513 | 11 | 5.52E-02 | 3.18 | 7.32E-07 |
| *rs2157719 | | *rs4977756 | 12 | 7.08E-02 | 4.86 | 9.23E-30 |
| *rs2157719 | | #rs7530361 | 11 | 5.51E-02 | 3.20 | 6.83E-07 |
| *rs2157719 | | #rs506044 | 11 | 5.54E-02 | 3.12 | 9.64E-07 |
| *rs2157719 | | #rs640030 | 11 | 5.49E-02 | 3.16 | 8.12E-07 |
| *rs2157719 | | #rs687513 | 11 | 5.59E-02 | 3.07 | 1.35E-06 |
| *rs1063192 | | *rs4977756 | 12 | 7.63E-02 | 4.54 | 1.41E-28 |
| *rs1063192 | | #rs7530361 | 11 | 5.60E-02 | 3.12 | 1.13E-06 |
| *rs1063192 | | #rs506044 | 11 | 5.63E-02 | 3.05 | 1.60E-06 |
| *rs1063192 | | #rs640030 | 11 | 5.59E-02 | 3.08 | 1.35E-06 |
| *rs4977756 | | #rs7530361 | 11 | 5.35E-02 | 4.28 | 3.14E-10 |
| *rs4977756 | | #rs501700 | 11 | 5.44E-02 | 4.17 | 5.57E-10 |
| *rs4977756 | | #rs506044 | 11 | 5.36E-02 | 4.18 | 4.46E-10 |
| *rs4977756 | | #rs640030 | 11 | 5.31E-02 | 4.24 | 3.66E-10 |
| *rs4977756 | | #rs687513 | 11 | 5.41E-02 | 4.10 | 6.86E-10 |
| rs2736100 | #rs7530361 | rs1920116 | 212 | 5.01E-02 | 4.30 | 5.02E-10 |
| *rs1412829 | *rs2157719 | *rs4977756 | 112 | 6.91E-02 | 5 | 3.63E-30 |
| *rs1412829 | *rs2157719 | #rs7530361 | 111 | 5.43E-02 | 3.23 | 6.64E-07 |
| *rs1412829 | *rs2157719 | #rs501700 | 111 | 5.49E-02 | 3.03 | 2.87E-06 |
| *rs1412829 | *rs2157719 | #rs506044 | 111 | 5.45E-02 | 3.15 | 9.54E-07 |
| *rs1412829 | *rs2157719 | #rs640030 | 111 | 5.41E-02 | 3.19 | 7.97E-07 |
| *rs1412829 | *rs2157719 | #rs687513 | 111 | 5.50E-02 | 3.1 | 1.35E-06 |
| *rs1412829 | *rs1063192 | *rs4977756 | 112 | 6.73E-02 | 5.13 | 3.30E-30 |
| *rs1412829 | *rs1063192 | #rs7530361 | 111 | 5.40E-02 | 3.27 | 5.38E-07 |
| *rs1412829 | *rs1063192 | #rs501700 | 111 | 5.46E-02 | 3.06 | 2.39E-06 |
| *rs1412829 | *rs1063192 | #rs506044 | 111 | 5.42E-02 | 3.19 | 7.58E-07 |
| *rs1412829 | *rs1063192 | #rs640030 | 111 | 5.38E-02 | 3.23 | 6.33E-07 |
| *rs1412829 | *rs1063192 | #rs687513 | 111 | 5.47E-02 | 3.14 | 1.08E-06 |
| *rs1412829 | *rs4977756 | #rs7530361 | 122 | 5.80E-02 | 5.61 | 5.10E-26 |
| *rs1412829 | *rs4977756 | #rs501700 | 122 | 5.83E-02 | 5.61 | 2.95E-26 |
| *rs1412829 | *rs4977756 | rs1920116 | 122 | 5.28E-02 | 5.56 | 1.55E-23 |
| *rs1412829 | *rs4977756 | #rs506044 | 122 | 5.79E-02 | 5.61 | 5.05E-26 |
| *rs1412829 | *rs4977756 | #rs640030 | 122 | 5.79E-02 | 5.61 | 5.07E-26 |
| *rs1412829 | *rs4977756 | #rs687513 | 122 | 5.79E-02 | 5.61 | 5.05E-26 |
| *rs1412829 | *rs4977756 | rs3779505 | 122 | 6.39E-02 | 5.14 | 1.60E-27 |
| *rs1412829 | #rs7530361 | #rs501700 | 111 | 5.47E-02 | 3.31 | 3.53E-07 |
| *rs1412829 | #rs7530361 | #rs506044 | 111 | 5.35E-02 | 3.33 | 3.05E-07 |
| *rs1412829 | #rs7530361 | #rs640030 | 111 | 5.37E-02 | 3.31 | 3.64E-07 |
| *rs1412829 | #rs7530361 | #rs687513 | 111 | 5.37E-02 | 3.32 | 3.34E-07 |
| *rs1412829 | #rs501700 | #rs506044 | 111 | 5.44E-02 | 3.26 | 4.44E-07 |
| *rs1412829 | #rs501700 | #rs640030 | 111 | 5.44E-02 | 3.26 | 4.74E-07 |
| *rs1412829 | #rs501700 | #rs687513 | 111 | 5.45E-02 | 3.24 | 4.96E-07 |
| *rs1412829 | #rs506044 | #rs640030 | 111 | 5.42E-02 | 3.29 | 3.82E-07 |
| *rs1412829 | #rs506044 | #rs687513 | 111 | 5.48E-02 | 3.23 | 5.39E-07 |
| *rs1412829 | #rs640030 | #rs687513 | 111 | 5.44E-02 | 3.27 | 4.22E-07 |
| *rs2157719 | *rs1063192 | *rs4977756 | 112 | 6.81E-02 | 5.02 | 6.23E-30 |
| *rs2157719 | *rs1063192 | #rs7530361 | 111 | 5.46E-02 | 3.17 | 9.01E-07 |
| *rs2157719 | *rs1063192 | #rs506044 | 111 | 5.49E-02 | 3.1 | 1.27E-06 |
| *rs2157719 | *rs1063192 | #rs640030 | 111 | 5.44E-02 | 3.14 | 1.07E-06 |
| *rs2157719 | *rs1063192 | #rs687513 | 111 | 5.54E-02 | 3.05 | 1.78E-06 |
| *rs2157719 | *rs4977756 | #rs7530361 | 122 | 5.83E-02 | 5.33 | 4.74E-25 |
| *rs2157719 | *rs4977756 | #rs501700 | 122 | 5.86E-02 | 5.34 | 2.76E-25 |
| *rs2157719 | *rs4977756 | rs1920116 | 122 | 5.27E-02 | 5.31 | 9.62E-23 |
| *rs2157719 | *rs4977756 | #rs506044 | 122 | 5.82E-02 | 5.34 | 4.68E-25 |
| *rs2157719 | *rs4977756 | #rs640030 | 122 | 5.82E-02 | 5.34 | 4.70E-25 |
| *rs2157719 | *rs4977756 | #rs687513 | 122 | 5.82E-02 | 5.34 | 4.68E-25 |
| *rs2157719 | *rs4977756 | rs3779505 | 122 | 6.39E-02 | 4.94 | 1.17E-26 |
| *rs2157719 | #rs7530361 | #rs501700 | 111 | 5.54E-02 | 3.2 | 6.57E-07 |
| *rs2157719 | #rs7530361 | #rs506044 | 111 | 5.41E-02 | 3.22 | 5.73E-07 |
| *rs2157719 | #rs7530361 | #rs640030 | 111 | 5.44E-02 | 3.19 | 6.80E-07 |
| *rs2157719 | #rs7530361 | #rs687513 | 111 | 5.43E-02 | 3.2 | 6.25E-07 |
| *rs2157719 | #rs501700 | #rs506044 | 111 | 5.50E-02 | 3.15 | 8.19E-07 |
| *rs2157719 | #rs501700 | #rs640030 | 111 | 5.50E-02 | 3.15 | 8.91E-07 |
| *rs2157719 | #rs501700 | #rs687513 | 111 | 5.52E-02 | 3.14 | 9.10E-07 |
| *rs2157719 | #rs506044 | #rs640030 | 111 | 5.49E-02 | 3.18 | 7.09E-07 |
| *rs2157719 | #rs506044 | #rs687513 | 111 | 5.54E-02 | 3.12 | 9.86E-07 |
| *rs2157719 | #rs640030 | #rs687513 | 111 | 5.51E-02 | 3.16 | 7.78E-07 |
| *rs1063192 | *rs4977756 | #rs7530361 | 122 | 6.25E-02 | 4.95 | 3.66E-24 |
| *rs1063192 | *rs4977756 | #rs501700 | 122 | 6.29E-02 | 4.94 | 2.27E-24 |
| *rs1063192 | *rs4977756 | rs1920116 | 122 | 5.68E-02 | 4.74 | 1.32E-21 |
| *rs1063192 | *rs4977756 | #rs506044 | 122 | 6.23E-02 | 4.95 | 3.51E-24 |
| *rs1063192 | *rs4977756 | #rs640030 | 122 | 6.23E-02 | 4.95 | 3.54E-24 |
| *rs1063192 | *rs4977756 | #rs687513 | 122 | 6.24E-02 | 4.95 | 3.54E-24 |
| *rs1063192 | *rs4977756 | rs3779505 | 122 | 6.88E-02 | 4.59 | 1.30E-25 |
| *rs1063192 | #rs7530361 | #rs501700 | 111 | 5.63E-02 | 3.12 | 1.09E-06 |
| *rs1063192 | #rs7530361 | #rs506044 | 111 | 5.51E-02 | 3.13 | 9.58E-07 |
| *rs1063192 | #rs7530361 | #rs640030 | 111 | 5.53E-02 | 3.11 | 1.13E-06 |
| *rs1063192 | #rs7530361 | #rs687513 | 111 | 5.52E-02 | 3.12 | 1.04E-06 |
| *rs1063192 | #rs501700 | #rs506044 | 111 | 5.59E-02 | 3.07 | 1.36E-06 |
| *rs1063192 | #rs501700 | #rs640030 | 111 | 5.60E-02 | 3.07 | 1.50E-06 |
| *rs1063192 | #rs501700 | #rs687513 | 111 | 5.61E-02 | 3.06 | 1.51E-06 |
| *rs1063192 | #rs506044 | #rs640030 | 111 | 5.58E-02 | 3.1 | 1.19E-06 |
| *rs1063192 | #rs506044 | #rs687513 | 111 | 5.64E-02 | 3.04 | 1.64E-06 |
| *rs1063192 | #rs640030 | #rs687513 | 111 | 5.60E-02 | 3.08 | 1.30E-06 |
| *rs4977756 | #rs7530361 | #rs501700 | 111 | 5.36E-02 | 4.28 | 2.86E-10 |
| *rs4977756 | #rs7530361 | #rs506044 | 111 | 5.22E-02 | 4.33 | 2.22E-10 |
| *rs4977756 | #rs7530361 | #rs640030 | 111 | 5.25E-02 | 4.29 | 2.81E-10 |
| *rs4977756 | #rs7530361 | #rs687513 | 111 | 5.24E-02 | 4.3 | 2.55E-10 |
| *rs4977756 | #rs501700 | #rs506044 | 111 | 5.31E-02 | 4.23 | 3.36E-10 |
| *rs4977756 | #rs501700 | #rs640030 | 111 | 5.31E-02 | 4.24 | 3.70E-10 |
| *rs4977756 | #rs501700 | #rs687513 | 111 | 5.33E-02 | 4.2 | 3.88E-10 |
| *rs4977756 | #rs506044 | #rs640030 | 111 | 5.30E-02 | 4.26 | 2.95E-10 |
| *rs4977756 | #rs506044 | #rs687513 | 111 | 5.36E-02 | 4.18 | 4.47E-10 |
| *rs4977756 | #rs640030 | #rs687513 | 111 | 5.32E-02 | 4.24 | 3.32E-10 |

+ 1 is the minor allele (risk allele)

# SNP on chromosome 1

* SNP on chromosome 9 in gene CDKN2A/B

Pvalue_OR_ is the asymptotic pvalue for the Odds Ratio estimated using Wald test.
