# Supplementary material for: Combinations of newly confirmed Glioma-Associated loci link regions on chromosomes 1 and 9 to increased disease risk
Source: BMC Med Genomics. 2011 Aug 9;4:63. doi: 10.1186/1755-8794-4-63 (PMC3212919; doi:10.1186/1755-8794-4-63)
Supplement: Additional file 4 — Table S4. KEGG pathways with SNPs (p < 0.001) from both AGS and TCGA studies. Boldface denotes pathways with significant genes that are common to both populations. [file 1755-8794-4-63-S4.DOC]

Table S4. KEGG pathways with SNPs ( p < 0.001) from both AGS and TCGA studies. Boldface denotes pathways with significant genes that are common to both populations.

| PATHWAY | TCGA | GENE | UCSF | GENE |
| --- | --- | --- | --- | --- |
| Metabolic pathways | rs12416497 | ADK | rs12021720 | DBT |
|  | rs9932597 | TK2 | rs2810424 | DBT |
|  | rs2411161 | ACACA | rs60848849 | GALNT14 |
|  | rs2514722 | EXT1 | rs1980444 | PLA2G4A |
|  | rs3807552 | DDC |  |  |
|  | rs3829897 | DDC |  |  |
|  | rs6788652 | PLD1 |  |  |
|  | rs7074861 | GPAM |  |  |
|  | rs7587303 | SCLY |  |  |
|  | rs7795944 | ATP6V0E2 |  |  |
|  | rs990282 | TPK1 |  |  |
| **Purine metabolism** | rs12416497 | ADK | rs7617530 | FHIT |
|  | rs13059601 | FHIT |  |  |
|  | rs1347732 | PDE1A |  |  |
|  | rs1874309 | PDE1B |  |  |
|  | rs295936 | PDE4D |  |  |
|  | rs7592850 | PDE1A |  |  |
|  | rs9365900 | PDE10A |  |  |
| Glycerophospholipid metabolism | rs6788652 | PLD1 | rs1980444 | PLA2G4A |
|  | rs7074861 | GPAM |  |  |
| Ether lipid metabolism | rs6788652 | PLD1 | rs1980444 | PLA2G4A |
| MAPK signaling pathway | rs11886908 | CACNB4 | rs140040 | CACNG2 |
|  | rs11902858 | CACNB4 | rs1344307 | EGFR |
|  | rs1476868 | BRAF | rs4791036 | PRKCA |
|  | rs3816027 | CACNA1A | rs208346 | GNA12 |
|  | rs4710123 | RPS6KA2 | rs1980444 | PLA2G4A |
|  | rs7011216 | STK3 |  |  |
| ErbB signaling pathway | rs1476868 | BRAF | rs1344307 | EGFR |
|  | rs16986333 | NA | rs4791036 | PRKCA |
|  | rs7883013 | NA |  |  |
|  | rs916873 | CAMK2D |  |  |
| Calcium signaling pathway | rs1347732 | PDE1A | rs1344307 | EGFR |
|  | rs1799286 | GRIN2D | rs4791036 | PRKCA |
|  | rs1874309 | PDE1B |  |  |
|  | rs2676077 | RYR3 |  |  |
|  | rs3816027 | CACNA1A |  |  |
|  | rs7592850 | PDE1A |  |  |
|  | rs916873 | CAMK2D |  |  |
| ytokine-cytokine receptor interaction | rs1359837 | EDA | rs10079250 | CSF1R |
|  | rs7552086 | IL28RA | rs216148 | CSF1R |
|  |  |  | rs1344307 | EGFR |
| hemokine signaling pathway | rs1476868 | BRAF | rs6066856 | PREX1 |
|  |  |  | rs3746816 | PREX1 |
| **Neuroactive ligand-receptor interaction** | rs12904325 | GABRG3 | rs1011455 | GABRG3 |
|  | rs17539361 | GABRB1 | rs3864070 | GRM7 |
|  | rs1799286 | GRIN2D | rs4887546 | GABRG3 |
|  | rs1815771 | GRIK4 | rs1011456 | GABRG3 |
|  | rs1930409 | GABBR2 |  |  |
|  | rs324041 | DRD3 |  |  |
|  | rs513823 | GRIK3 |  |  |
|  | rs7658410 | GABRA4 |  |  |
| Cell cycle | rs10113111 | RAD21 | rs1063192 | CDKN2B |
|  |  |  | rs3217992 | CDKN2B |
| Oocyte meiosis | rs2946834 | IGF1 | rs805674 | SLK |
|  | rs4710123 | RPS6KA2 | rs3740469 | SLK |
|  | rs916873 | CAMK2D |  |  |
| Endocytosis | rs10886193 | RAB11FIP2 | rs10079250 | CSF1R |
|  | rs16896742 | NA | rs216148 | CSF1R |
|  | rs4609234 | DDEF1 | rs1344307 | EGFR |
|  | rs6788652 | PLD1 | rs1386689 | PSD3 |
|  | rs9694314 | DDEF1 | rs2638648 | PSD3 |
|  |  |  | rs7975313 | IQSEC3 |
| Cardiac muscle contraction | rs11886908 | CACNB4 | rs140040 | CACNG2 |
|  | rs11902858 | CACNB4 | rs1531136 | MYL3 |
| **Vascular smooth muscle contraction** | rs1476868 | BRAF | rs4400745 | PRKG1 |
|  | rs1922139 | PRKG1 | rs4466778 | PRKG1 |
|  |  |  | rs4791036 | PRKCA |
|  |  |  | rs208346 | GNA12 |
|  |  |  | rs1980444 | PLA2G4A |
| Wnt signaling pathway | rs10483712 | DAAM1 | rs4791036 | PRKCA |
|  | rs11861500 | NFAT5 |  |  |
|  | rs1252915 | DAAM1 |  |  |
|  | rs2295849 | DAAM1 |  |  |
|  | rs6573252 | DAAM1 |  |  |
|  | rs7143698 | DAAM1 |  |  |
|  | rs916873 | CAMK2D |  |  |
| **Axon guidance** | rs11082983 | DCC | rs12341266 | RGS3 |
|  | rs11813036 | ABLIM1 | rs1145245 | DCC |
|  | rs11861500 | NFAT5 |  |  |
|  | rs11872471 | DCC |  |  |
|  | rs12604940 | DCC |  |  |
|  | rs16986333 | NA |  |  |
|  | rs2244140 | UNC5B |  |  |
|  | rs2288794 | SLIT3 |  |  |
|  | rs3748735 | PLXNA2 |  |  |
|  | rs6820369 | UNC5C |  |  |
|  | rs7373232 | EPHA3 |  |  |
|  | rs7883013 | NA |  |  |
|  | rs881758 | PLXNA2 |  |  |
| VEGF signaling pathway | rs11861500 | NFAT5 | rs4791036 | PRKCA |
|  |  |  | rs1980444 | PLA2G4A |
| **Focal adhesion** | rs11017181 | DOCK1 | rs3779505 | ITGB8 |
|  | rs12146386 | DOCK1 | rs2301727 | ITGB8 |
|  | rs1476868 | BRAF | rs3807936 | ITGB8 |
|  | rs16952065 | ITGA11 | rs2158250 | ITGB8 |
|  | rs16986333 | NA | rs1344307 | EGFR |
|  | rs2274617 | ITGA10 | rs4791036 | PRKCA |
|  | rs9492200 | LAMA2 | rs7574757 | COL4A4 |
|  | rs2720986 | DOCK1 | rs2454585 | ITGA1 |
|  | rs2766034 | DOCK1 | rs2277084 | LAMA4 |
|  | rs2946834 | IGF1 |  |  |
|  | rs3779505 | ITGB8 |  |  |
|  | rs7614116 | COL6A6 |  |  |
|  | rs7883013 | NA |  |  |
|  | rs895135 | ITGA11 |  |  |
| **ECM-receptor interaction** | rs16952065 | ITGA11 | rs3779505 | ITGB8 |
|  | rs2274617 | ITGA10 | rs2301727 | ITGB8 |
|  | rs3779505 | ITGB8 | rs3807936 | ITGB8 |
|  | rs7614116 | COL6A6 | rs2158250 | ITGB8 |
|  | rs895135 | ITGA11 | rs7574757 | COL4A4 |
|  | rs9492200 | LAMA2 | rs2454585 | ITGA1 |
|  |  |  | rs2277084 | LAMA4 |
| **Cell adhesion molecules (CAMs)** | rs16896742 | NA | rs3779505 | ITGB8 |
|  | rs2254911 | CD86 | rs2301727 | ITGB8 |
|  | rs3779505 | ITGB8 | rs3807936 | ITGB8 |
|  | rs661059 | PTPRM | rs2158250 | ITGB8 |
|  | rs7710491 | VCAN |  |  |
| Adherens junction | rs661059 | PTPRM | rs1344307 | EGFR |
|  | rs7587388 | CTNNA2 | rs11615170 | PTPRB |
|  | rs890861 | INSR |  |  |
| Tight junction | rs16838654 | PPP2R2C | rs4791036 | PRKCA |
|  | rs2672734 | EXOC3 |  |  |
|  | rs7218683 | MYH10 |  |  |
|  | rs7587388 | CTNNA2 |  |  |
| **Gap junction** | rs1922139 | PRKG1 | rs4400745 | PRKG1 |
|  |  |  | rs4466778 | PRKG1 |
|  |  |  | rs1344307 | EGFR |
|  |  |  | rs4791036 | PRKCA |
| Natural killer cell mediated cytotoxicity | rs11861500 | NFAT5 | rs4791036 | PRKCA |
|  | rs1476868 | BRAF |  |  |
| Fc gamma R-mediated phagocytosis | rs10760169 | GSN | rs4791036 | PRKCA |
|  | rs4609234 | DDEF1 | rs4427071 | AMPH |
|  | rs6788652 | PLD1 | rs1980444 | PLA2G4A |
|  | rs9694314 | DDEF1 |  |  |
| Leukocyte transendothelial migration | rs7587388 | CTNNA2 | rs4791036 | PRKCA |
| Long-term potentiation | rs1476868 | BRAF | rs4791036 | PRKCA |
|  | rs1799286 | GRIN2D |  |  |
|  | rs4710123 | RPS6KA2 |  |  |
|  | rs916873 | CAMK2D |  |  |
| **Long-term depression** | rs1476868 | BRAF | rs4400745 | PRKG1 |
|  | rs1922139 | PRKG1 | rs4466778 | PRKG1 |
|  | rs2946834 | IGF1 | rs4791036 | PRKCA |
|  | rs3816027 | CACNA1A | rs208346 | GNA12 |
|  |  |  | rs1980444 | PLA2G4A |
| **Olfactory transduction** | rs12278907 | OR2AG2 | rs4400745 | PRKG1 |
|  | rs1922139 | PRKG1 | rs7145814 | OR5AU1 |
|  | rs2775257 | OR4K13 | rs4466778 | PRKG1 |
|  | rs916873 | CAMK2D | rs7555310 | OR2M7 |
| **Regulation of actin cytoskeleton** | rs10760169 | GSN | rs3779505 | ITGB8 |
|  | rs11017181 | DOCK1 | rs2301727 | ITGB8 |
|  | rs12146386 | DOCK1 | rs3807936 | ITGB8 |
|  | rs12822067 | NCKAP1L | rs2158250 | ITGB8 |
|  | rs1476868 | BRAF | rs1344307 | EGFR |
|  | rs16952065 | ITGA11 | rs208346 | GNA12 |
|  | rs895135 | ITGA11 | rs2454585 | ITGA1 |
|  | rs2036068 | NCKAP1L |  |  |
|  | rs2274617 | ITGA10 |  |  |
|  | rs7218683 | MYH10 |  |  |
|  | rs2720986 | DOCK1 |  |  |
|  | rs2766034 | DOCK1 |  |  |
|  | rs3779505 | ITGB8 |  |  |
| GnRH signaling pathway | rs6788652 | PLD1 | rs1344307 | EGFR |
|  | rs916873 | CAMK2D | rs4791036 | PRKCA |
|  |  |  | rs1980444 | PLA2G4A |
| Melanogenesis | rs916873 | CAMK2D | rs4791036 | PRKCA |
| Alzheimer's disease | rs16947481 | ERN1 | rs2837990 | BACE2 |
|  | rs1799286 | GRIN2D |  |  |
|  | rs2676077 | RYR3 |  |  |
| Vibrio cholerae infection | rs7795944 | ATP6V0E2 | rs4791036 | PRKCA |
| Epithelial cell signaling  in Helicobacter pylori infection | rs7795944 | ATP6V0E2 | rs1344307 | EGFR |
| **Pathways in cancer** | rs11082983 | DCC | rs1063192 | CDKN2B |
|  | rs11191310 | SUFU | rs10079250 | CSF1R |
|  | rs11872471 | DCC | rs216148 | CSF1R |
|  | rs12604940 | DCC | rs3217992 | CDKN2B |
|  | rs1476868 | BRAF | rs1344307 | EGFR |
|  | rs9492200 | LAMA2 | rs4791036 | PRKCA |
|  | rs2659122 | KLK3 | rs7574757 | COL4A4 |
|  | rs2946834 | IGF1 | rs2277084 | LAMA4 |
|  | rs6788652 | PLD1 | rs12723208 | TRAF5 |
|  | rs7587388 | CTNNA2 | rs1145245 | DCC |
| **Colorectal cancer** | rs11082983 | DCC | rs1344307 | EGFR |
|  | rs11872471 | DCC | rs1145245 | DCC |
|  | rs12604940 | DCC |  |  |
|  | rs1476868 | BRAF |  |  |
| Pancreatic cancer | rs1476868 | BRAF | rs1344307 | EGFR |
|  | rs6788652 | PLD1 |  |  |
| Endometrial cancer | rs1476868 | BRAF | rs1344307 | EGFR |
|  | rs7587388 | CTNNA2 |  |  |
| Glioma | rs1476868 | BRAF | rs1344307 | EGFR |
|  | rs916873 | CAMK2D | rs4791036 | PRKCA |
|  | rs2946834 | IGF1 |  |  |
| Prostate cancer | rs1476868 | BRAF | rs1344307 | EGFR |
|  | rs2659122 | KLK3 |  |  |
|  | rs2946834 | IGF1 |  |  |
| elanoma | rs1476868 | BRAF | rs1344307 | EGFR |
|  | rs2946834 | IGF1 |  |  |
| Bladder cancer | rs1476868 | BRAF | rs1344307 | EGFR |
| **Small cell lung cancer** | rs13059601 | FHIT | rs1063192 | CDKN2B |
|  | rs9492200 | LAMA2 | rs3217992 | CDKN2B |
|  |  |  | rs7617530 | FHIT |
|  |  |  | rs7574757 | COL4A4 |
|  |  |  | rs2277084 | LAMA4 |
|  |  |  | rs12723208 | TRAF5 |
| **Non-small cell lung cancer** | rs13059601 | FHIT | rs1344307 | EGFR |
|  | rs1476868 | BRAF | rs4791036 | PRKCA |
|  |  |  | rs7617530 | FHIT |
| **Hypertrophic cardiomyopathy (HCM)** | rs11886908 | CACNB4 | rs3779505 | ITGB8 |
|  | rs11902858 | CACNB4 | rs2301727 | ITGB8 |
|  | rs16952065 | ITGA11 | rs140040 | CACNG2 |
|  | rs2274617 | ITGA10 | rs3807936 | ITGB8 |
|  | rs2742347 | TTN | rs2158250 | ITGB8 |
|  | rs2946834 | IGF1 | rs1531136 | MYL3 |
|  | rs3779505 | ITGB8 | rs2454585 | ITGA1 |
|  | rs9492200 | LAMA2 |  |  |
|  | rs895135 | ITGA11 |  |  |
| **Arrhythmogenic right ventricular**  **Cardiomyopathy** | rs1046116 | PKP2 | rs3779505 | ITGB8 |
|  | rs11886908 | CACNB4 | rs2301727 | ITGB8 |
|  | rs11902858 | CACNB4 | rs140040 | CACNG2 |
|  | rs16952065 | ITGA11 | rs3807936 | ITGB8 |
|  | rs2274617 | ITGA10 | rs2158250 | ITGB8 |
|  | rs3779505 | ITGB8 | rs2454585 | ITGA1 |
|  | rs7587388 | CTNNA2 |  |  |
|  | rs895135 | ITGA11 |  |  |
|  | rs9492200 | LAMA2 |  |  |
| **Dilated cardiomyopathy** | rs11886908 | CACNB4 | rs3779505 | ITGB8 |
|  | rs11902858 | CACNB4 | rs2301727 | ITGB8 |
|  | rs16952065 | ITGA11 | rs140040 | CACNG2 |
|  | rs2274617 | ITGA10 | rs3807936 | ITGB8 |
|  | rs2742347 | TTN | rs2158250 | ITGB8 |
|  | rs2946834 | IGF1 | rs1531136 | MYL3 |
|  | rs3779505 | ITGB8 | rs2454585 | ITGA1 |
